# Evaluation of IMERG and ERA5 precipitation products over the Mongolian Plateau

**DOI:** 10.1038/s41598-022-26047-8

**Published:** 2022-12-16

**Authors:** Ying Xin, Yaping Yang, Xiaona Chen, Xiafang Yue, Yangxiaoyue Liu, Cong Yin

**Affiliations:** 1 State Key Laboratory of Resources and Environmental Information System, Institute of Geographic Sciences and Natural Resources Research, Chinese Academy of Sciences, Beijing, China; 2 College of Resources and Environment, University of Chinese Academy of Sciences, Beijing, China; 3 Jiangsu Center for Collaborative Innovation in Geographical Information Resource Development and Application, Nanjing, China

**Keywords:** Climate and Earth system modelling, Hydrology, Hydrology

## Abstract

Precipitation is an important component of the hydrological cycle and has significant impact on ecological environment and social development, especially in arid areas where water resources are scarce. As a typical arid and semi-arid region, the Mongolian Plateau is ecologically fragile and highly sensitive to climate change. Reliable global precipitation data is urgently needed for the sustainable development over this gauge-deficient region. With high-quality estimates, fine spatiotemporal resolutions, and wide coverage, the state-of-the-art Integrated Multi-satellite Retrievals for Global Precipitation Measurement (IMERG) and European Center for Medium-range Weather Forecasts Reanalysis 5 (ERA5) have great potential for regional climatic, hydrological, and ecological applications. However, how they perform has not been well investigated on the Mongolian Plateau. Therefore, this study evaluated the performance of three IMERG V06 datasets (ER, LR and FR), two ERA5 products (ERA5-HRES and ERA5-Land), and their predecessors (TMPA-3B42 and ERA-Interim) over the region across 2001–2018. The results showed that all products broadly characterized seasonal precipitation cycles and spatial patterns, but only the three reanalysis products, IMERG FR and TMPA-3B42 could capture interannual and decadal variability. When describing daily precipitation, dataset performances ranked ERA5-Land > ERA5-HRES > ERA-Interim > IMERG FR > IMERG LR > IMERG ER > TMPA-3B42. All products showed deficiencies in overestimating weak precipitation and underestimating high-intensity precipitation. Besides, products performed best in agricultural lands and forests along the northern and south-eastern edges, followed by urban areas and grasslands closer to the center, and worst in the sparse vegetation and bare areas of the south-west. Due to a negative effect of topographic complexity, IMERG showed poor detection capabilities in forests. Accordingly, this research currently supports the applicability of reanalysis ERA5 data over the arid, topographically complex Mongolian Plateau, which can inform regional applications with different requirements.

## Introduction

Precipitation is an essential component of the global material and energy cycles^1^. High-quality precipitation data are vital to climate change research, environmental monitoring, water resources management, and disaster predictions^2–5^. To this end, ground-based rain gauge stations have long been the primary means of acquiring accurate precipitation measurements^6^, and international networks of rain gauges form the foundation of global precipitation observation systems^7,8^. However, this approach is constrained by the uneven distribution and limited spatial representation of stations^9,10^.

With refinements of numerical simulations and earth observation techniques, satellite and model-based approaches have emerged as effective methods for collecting accurate precipitation data with good spatiotemporal continuity. For example, based on extensive meteorological satellite observations, precipitation estimates can be obtained by visible, infrared, microwave, or multi-sensor joint inversion^11–14^. These approaches have produced a series of precipitation datasets, such as the Tropical Rainfall Measurement Mission (TRMM) Multi-satellite Precipitation Analysis (TMPA)^15^, Global Satellite Mapping of Precipitation^16^, and Climate Prediction Center Morphing Technique^14^. With high accuracy and fine spatial resolution, satellite precipitation products are widely used in regional hydrological^17,18^, ecological^19,20^, and agricultural^21^ applications. However, limited by the satellite launching times, these products can only provide estimates from the 1990s onwards. In comparison, model-based approaches, which generate precipitation estimates based on numerical simulations with relatively good geographical and physical consistency, can provide data with globally spatial coverage and decades of temporal coverage^22^. For example, atmospheric reanalysis optimally integrates observations with short-term numerical forecast model outputs via data assimilation, providing long-term, regular gridded products containing vertical atmospheric field information. To date, various reanalysis products have been used, including Japanese 55-year Reanalysis^23^, Climate Forecast System Reanalysis^24,25^, and the European Centre for Medium-range Weather Forecasts (ECMWF) ReAnalysis Interim (ERA-Interim)^26^. But the coarse spatial resolution and large uncertainties in complex terrain limit their ability to provide reliable precipitation information at fine scales^27,28^. Despite their respective deficiencies, satellite and reanalysis approaches still provide powerful techniques for obtaining information for regions lacking stations.

Among the numerous precipitation products available, the recently released Integrated Multi-satellite Retrievals for Global Precipitation Measurement (GPM) Mission (IMERG), and the European Center for Medium-range Weather Forecasts ReAnalysis 5 (ERA5) precipitation data comprise the most advanced satellite-based and reanalysis techniques, respectively. Compared to their predecessors, IMERG and ERA5 have substantially improved spatiotemporal coverages, resolutions, and product performances^29,30^. For example, the GPM Core Observatory satellite is equipped with the first dual-frequency precipitation radar with Ka-band (35.5 GHz), allowing IMERG to have better capability than TMPA when detecting weak and solid precipitation^31^. Produced based on advanced 4D-var data assimilation scheme and model forecasts in Cycle 41r2 version of the ECMWF Integrated Forecasting System (IFS)^32^, ERA5 provides more output parameters and uncertainty information compared to ERA-Interim^30^.

Since the release of these two datasets, numerous global researchers have evaluated their performance. For example, Tang et al. compared IMERG V04 (Final Run) and TMPA-3B42 V7 across mainland China, finding that although IMERG performed better, it required further improvements in arid areas^33^. Elsewhere, studies have noted that IMERG overestimates weak precipitation, and underestimates strong precipitation^34–36^. Many researchers have pointed out that topography also affects the estimation bias of IMERG, but where overestimations or underestimations occur (in the mountains or plains) varies across regions^37–39^. Evaluations have also been conducted in regions such as China^40,41^, India^42^, Canada^43^, and the United States^44^, which have recognized the improvements of IMERG over TMPA, while demonstrating its limitations in arid and high-latitude regions. For ERA5 precipitation, Zandler et al. noted that it was better at capturing spatiotemporal trends than estimating precipitation amounts in the Central Asian mountains^45^. An assessment in Australia found that ERA5 would overestimate (underestimate) weak (strong) precipitation frequency^46^. Amjad et al., investigated ERA5 performance in Turkey, revealing that the product performed worse in areas with more complex topography^47^. Similar studies in North America^48^, China^49^, India^50^, Iran^51^, East Africa^52^, and Europe^53^ have all recognized the ability of ERA5 to capture precipitation patterns, while reporting deficiencies in the product’s characterization of high-intensity rainfall under complex topography.

There also exist some comparative studies as well. For example, Beck et al. compared IMERG V05 and ERA5 across the conterminous United States, finding that the former performed better in areas dominated by convective storms, while the latter was superior under complex terrains^54^. Tang et al. concluded that IMERG generally outperformed ERA5 across China, and can better reproduce precipitation diurnal cycles^55^. Other studies were presented in Central Asia^56^, India^57^, Turkey^47^, Iran^58^, and the United States^59^. Most studies have shown that IMERG outperforms ERA5, but the superiority of each dataset varies by regions, precipitation intensity, and altitude.

As a typical arid and semi-arid area, the Mongolian Plateau is ecologically fragile and sensitive to climate change, which makes it one of the key regions for climate and ecological research^60^. Precipitation has a significant impact on the ecological environment and social development of the region. For example, precipitation is an important supply source for rivers and lakes in arid environments, providing the main available water resources for the region^61^. It also affects the growth and productivity of vegetation by changing soil moisture^62,63^, which further impacts the structure and function of ecosystems^64^. Adequate precipitation can improve the ecosystem quality, thus alleviating regional environmental problems such as drought, desertification, and dust storms^65^. In addition, sufficient precipitation is beneficial to the growth of crops and pastures, which can promote the development of local agriculture and animal husbandry^66,67^. Therefore, high-quality precipitation information is urgently needed for regional climate research, ecological protection, and developmental planning. Due to the sparse distribution of stations on the Mongolian Plateau, spatially continuous global data like IMERG and ERA5 holds great significance for studies at regional scales. However, detailed and systematic validations or comparisons of the IMERG and ERA5 datasets are still lacking on the Mongolian Plateau to show their strengths and weaknesses for different regional applications. In addition, most prior studies have focused on the product ability to estimate precipitation amount or detect precipitation occurrence, rather than investigating their performance in capturing precipitation patterns at different spatiotemporal scales. Nevertheless, research on climate change, extreme events, and ecological monitoring primarily require accurate precipitation variability, while smaller estimation error magnitudes are of secondary importance. Moreover, most evaluations for ERA5 have been carried out for the 0.25° dataset (ERA5-HRES), while few have investigated the higher resolution ERA5 land portion (ERA5-Land; 0.1°).

To explore the above issues, this study assessed and compared the performances of three IMERG (V06 Early Run, Late Run, and Final Run) and two ERA5 (ERA5-Land and ERA5-HRES) precipitation products, as well as their predecessors (TMPA-3B42 V7 and ERA-Interim), from 2001 to 2018 across the Mongolian Plateau. The evaluation was conducted to answer the following main questions: (1) whether IMERG and ERA5 can capture the spatial and temporal patterns of precipitation, and (2) how do they perform in estimating and detecting daily precipitation. The findings can inform advantages and remaining weaknesses of IMERG and ERA5 over the Mongolian Plateau, while serving as a reference for research and application data selection by regional needs.

## Study area and datasets

### Study area

**Figure 1:**
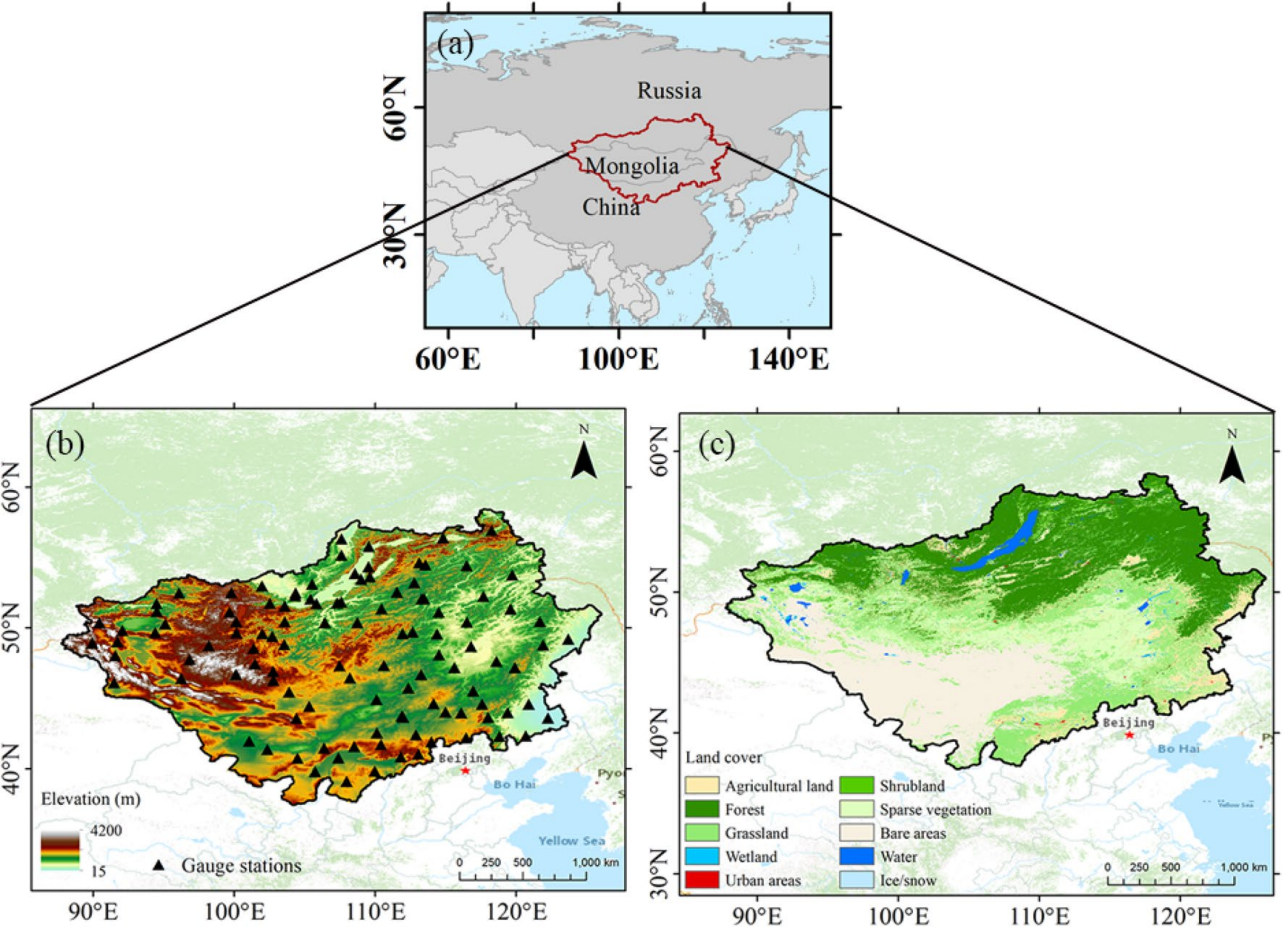
The (**a**) location, (**b**) topography and positions of the rain gauge stations, and (**c**) land cover of the Mongolian Plateau. The figure was created using ArcGIS 10.7 (https://www.esri.com/en-us/arcgis/about-arcgis/overview)^70^.

Located in the hinterland of Eurasia, the Mongolian Plateau contains the entirety of Mongolia, the Inner Mongolia Autonomous Region of China, as well as the Tuva Republic, Buryatia Republic, Zabaikalsky Krai, and parts of Irkutsk Oblast of Russia. Covering a total area of 3.82 million km^2^ between 85.75°–129° E, and 36°–59.75° N (Fig. 1), the region has an elevation of 15–4200 m, with the terrain gradually decreasing from west to east. Under the combined influence of westerly and East Asian monsoon circulations, the Mongolian Plateau is highly sensitive to climate change.

The study area is characterized by a temperate continental climate, where the average annual temperature ranges from ~ − 26 to 17 °C, while the average annual precipitation is < 200 mm in most areas (except in the eastern, northern, and northeastern parts, where > 400 mm is possible)^68^. Precipitation is concentrated in the summer (June–August), with the moisture mainly coming from the transport of westerlies^69^. Influenced by the Arctic Ocean to the north and Pacific Ocean to the east, precipitation gradually decreases from north to south and east to west with the decline of coastal proximity, reaching a minimum in the southwestern Gobi desert area.

### Datasets

#### Reference data

Global Surface Summary of the Day (GSOD) is a worldwide daily meteorological dataset produced by the National Climate Data Center of the National Oceanic and Atmospheric Administration, which is derived from the integrated surface hourly data. There were 146 stations located within the study area, 110 of which were valid stations (missing and invalid observations < 1% of the study period length—6574 days). These reference valid stations were relatively evenly distributed across the study area (Fig. 1b). The ground-based observations can be accessed through the National Centers for Environmental Information data website^71^.

#### Satellite precipitation products

Three IMERG V06 products, and one TMPA V7 product were used in the present analysis. The precipitation estimates of these satellite data are the sum of all forms of precipitation, including rain, drizzle, snow, gravel and hail^29,72^. As the predecessor of IMERG, TMPA is a representative precipitation dataset of TRMM jointly sponsored by the National Aeronautics and Space Administration (NASA) and the Japan Aerospace Exploration Agency. It is one of the most influential gridded precipitation data for nearly two decades, but no longer available after 2019. TMPA maintains a 3 h time step with a 0.25° spatial resolution across 50° N–50° S^73^. Its estimates are provided in two versions of real-time (TMPA-3B42RT) and post real-time (TMPA-3B42)^74^, with the former coming directly from calibrated passive microwave and infrared data, and the latter is bias-adjusted using monthly precipitation observations from the Global Precipitation Climate Center (GPCC) stations. In the present study, the daily TMPA-3B42 V7 dataset was selected as the representative predecessor of IMERG. This product is generated by simple summation of valid 3-hourly retrievals for the data day. Since the 3-hourly source data are in unit of mm/hr, a factor of 3 is applied to the sum. Notably, if a grid does not have valid retrievals on a given day, the corresponding daily gridded estimate will be set to a filled value^75^. The TMPA-3B42 data are available through the Goddard Earth Sciences Data and Information Services Center website^75^.

IMERG represents NASA’s newest satellite precipitation collection, with a fine spatial resolution of 0.1° and temporal resolution of 0.5 h^31^. It not only continues the TMPA services (covering June 2000 to present), but has greater spatial coverage (60° N–60° S) as well. This satellite product has three runs: Early Run (ER), Late Run (LR), and Final Run (FR), released 4 h, 12 h, and 3.5 months after observations, respectively^9^. Accordingly, the former two are near real-time products that can be used for time-sensitive applications (e.g., disaster warning), with the main difference being that ER use only forward morphing, while LR uses both forward and backward morphing. Research-level FR is calibrated using monthly GPCC data, and thus delivers more accurate precipitation information^76^. Here, all the three runs of IMERG V06 daily products derived from the same-day accumulation of valid half-hourly estimates were used for analysis. The calculation of daily accumulations is similar to that of the daily TMPA, except that a factor of 0.5 is applied to the sum^77^. The IMERG products can be found at the Precipitation Measurement Missions (PMM) website^78^.

#### Reanalysis precipitation products

Two advanced ERA5 datasets (ERA5-Land and ERA5-HRES) and their predecessor ERA-Interim were employed for evaluation. Here, the precipitation estimates of these reanalysis products are the accumulated liquid and frozen water falling on the Earth’s surface, depicting the sum of large-scale and convective precipitation^79–81^.

ERA-Interim is the fourth generation of atmospheric reanalysis dataset of the ECMWF. Based on the Cycle 31r2 of the IFS, it provides atmospheric data on 60 vertical levels, as well as 37 pressure, 16 potential temperature, and 1 potential vorticity level (s) via interpolation^26^. ERA-Interim provides 3-hourly information on global surface precipitation from 1979 to August 2019 with a spatial resolution of 0.75°^79^. The data can be acquired from the ECMWF website^82^.

Compared to ERA-Interim, ERA5 has improvements including a better 4D-var data assimilation scheme, more advanced prediction models, finer spatiotemporal resolutions, and three-hour uncertainty information^30^. Among the two products, ERA5-HRES refers to the high resolution realisation contained in ERA5 dataset, with a spatial resolution of 0.25° and a temporal resolution of 1 h. ERA5-Land is the land portion of ERA5 reanalysis data, which provides more accurate land parameters and better characterizes land status with its advanced 0.1° resolution. Another important difference between the two products is that ERA5-Land is forced only by the ERA5 low atmosphere meteorological field in conjunction with an additional lapse-rate correction, which is designed to make running ERA5‑Land computationally affordable^83^. Compared to ERA5-HRES, ERA5-Land is not coupled to the atmospheric module or ocean wave model of the ECMWF IFS, and runs without data assimilation. Notably, the hourly records provided by ERA5-HERS are accumulated precipitation over the hour ending at the validity time, while the ERA5-Land records are accumulated precipitation from 00 UTC to the hour ending at the forecast step^84^. The two ERA5 datasets are available on the Climate Data Store^80,81^.

#### Digital elevation model

The Advanced Spaceborne Thermal Emission and Reflection Radiometer (ASTER) Global Digital Elevation Model Version 3 (ASTGTMv003) was used to obtain elevation and slope information for the Mongolian Plateau. Developed by NASA in collaboration with the Ministry of Economy, Trade and Industry of Japan, ASTGTMv003 provides a digital elevation model (DEM) at 1 arc-second (~ 30 m at the equator), covering all land between 83° N and 83° S. Based on the entire ASTER Level 1A scenes from March 1, 2000 to November 30, 2013, DEMs were generated and cloud-masked using stereo correlation. All cloud-masked and non-cloud masked DEMs were stacked to produce ASTGTMv003, during which algorithms were used to remove bad values and outliers^85–87^. In addition, in regions where the number of stacked DEMs is limited, pre-existing reference DEMs were used for correcting residual anomalies. The DEM data can be downloaded from the Land Processes Distributed Active Archive Center website^88^.

#### Land cover

To investigate product performance under different land cover types, the European Space Agency (ESA) Climate Change Initiative Land Cover (CCI-LC) dataset was used for evaluation. CCI-LC is a continuous long-term global land cover product with 28 years of data (1992–2019) at 300 m grid resolution. Based on the ESA GlobCover product, it is produced by merging multiple earth observation data^89^, and divided into 22 global land cover classes (Table 1). For a clarity, we referred to the Intergovernmental Panel on Climate Change (IPCC) land categories and regrouped the CCI-LC classes into agricultural lands, forests, grasslands, wetlands, urban areas, shrubland, sparse vegetation, bare areas, water, and ice/snow. The land cover data can be acquired from the ESA CCI-LC website^90^.

**Table 1:** Correspondence between our regrouped land cover types and the CCI-LC legend.

Regrouped land cover types	Land cover classification system legend and corresponding code number used in the CCI-LC maps	
Agricultural land	10	Rainfed cropland
	20	Irrigated cropland
	30	Mosaic cropland (> 50%)/natural vegetation (tree, shrub, herbaceous cover) (< 50%)
	40	Mosaic natural vegetation (tree, shrub, herbaceous cover) (> 50%)/cropland (< 50%)
Forest	50	Tree cover, broadleaved, evergreen, closed to open (> 15%)
	60	Tree cover, broadleaved, deciduous, closed to open (> 15%)
	70	Tree cover, needleleaved, evergreen, closed to open (> 15%)
	80	Tree cover, needleleaved, deciduous, closed to open (> 15%)
	90	Tree cover, mixed leaf type (broadleaved and needleleaved)
	100	Mosaic tree and shrub (> 50%)/herbaceous cover (< 50%)
	160	Tree cover, flooded, fresh, or brackish water
	170	Tree cover, flooded, saline water
Grassland	110	Mosaic herbaceous cover (> 50%)/tree and shrub (< 50%)
	130	Grassland
Wetland	180	Shrub or herbaceous cover, flooded, fresh-saline, or brackish water
Urban areas	190	Urban
Shrubland	120	Shrubland
Sparse vegetation	140	Lichens and mosses
	150	Sparse vegetation (tree, shrub, herbaceous cover)
Bare areas	200	Bare areas
Water	210	Water
Ice/snow	220	Permanent snow and ice

## Methods

### Data pre-processing

To match the daily observations from ground-based stations, we first calculated the daily estimates of ERA-Interim, ERA5-HRES, and ERA5-Land using Eqs. (1)– (3), respectively, as follows^84^:

1 $${tp}_{d}=\left ({tp}_{12 00UTC}+{tp}_{12 12UTC}\right)\times 1000,$$

2 $${tp}_{d}=\left (\sum_{h=1}^{23}{tp}_{h}+{tp}_{d+1 00UTC}\right)\times 1000,$$

3 $${tp}_{d}={tp}_{d+1 00UTC}\times 1000,$$

where ${tp}_{d}$ is the daily precipitation estimate for a given day (unit in mm); ${tp}_{12 00UTC}$ and ${tp}_{12 12UTC}$ are ERA-Interim 3-hourly estimates of the day at the time step labelled 12, from time references 00UTC and 12UTC, respectively (unit in m); ${tp}_{h}$ is the ERA5 hourly record of the day (unit in m); and ${tp}_{d+1 00UTC}$ is the ERA5 hourly record at 00UTC on the next day (unit in m). Then, the daily satellite or reanalysis data were summed by month or year to obtained monthly and annual precipitation for evaluation for precipitation patterns.

Before evaluation, it is necessary to spatially match the point-wise station measurements and gridded estimates. To compare products with different spatial resolutions, many previous studies interpolated ground observations to grids and conducted pixel-to-pixel validation^41,55,91^. However, due to the sparseness of stations, station interpolation data cannot accurately represent the real precipitation situation over the Mongolian Plateau. Referring to existing evaluations in areas lacking stations^92–94^, we extracted gridded precipitation estimates for the corresponding stations with bilinear interpolation, and performed a pixel-to-point evaluation. The spatial matching approach would inevitably lead to bias in evaluation metric values^95^, which, nevertheless, would not have significant impact on our conclusions, since we focused more on the overall product performance than precise quantification of estimation errors in individual rainfall event. In addition, a previous research compared the techniques of pixel-to-pixel and pixel-to-point with bilinear interpolation, and found that they led to similar conclusions^96^.

To further analyze product ability to describe precipitation changes at longer temporal scales, the interannual and decadal variability of station and product data were calculated. Following some previous studies^94,97,98^, we obtained the interannual variability based on the normalized precipitation anomalies. For each precipitation record series (from gauge stations or product grids), monthly relative anomalies were calculated as the monthly anomaly divided by the corresponding monthly climatology. The relative anomalies for the 12 months within a year were then summed to obtain the normalized annual precipitation anomaly. The interannual variability of precipitation was calculated by removing decadal variation from the normalized annual anomaly. Notably, it has been found that the interannual spectral peak of precipitation over the Tibetan Plateau occurs at 3 and 10 year cycles^99^. Therefore, referring to the methods of Yuan et al., the decadal variability of precipitation was obtained by removing the interannual variability information through a 3-year running average^94^. Pearson correlation coefficients between the interannual/decadal variability for products and stations were used to indicate product ability to capture large-scale variability, and significance of the correlation coefficient was tested by the student’s t-test^99^.

### Evaluation metrics

To investigate the product ability to describe daily precipitation, we conducted the evaluation in terms of estimation accuracy and detection capability. A World Meteorological Organization (WMO) review on several evaluation scores^100^ recommended six common metrics for the present study: relative bias ($RB$), correlation coefficient ($Corr$), root mean square error ($RMSE$), probability of detection ($POD$), false alarm ratio ($FAR$), and equitable threat score ($ETS$), where the former three are accuracy metrics, and the latter three are detection metrics^101–103^. Accuracy metrics were used to measure the precision of estimates from the satellite and reanalysis products. $RB$ depicts the deviation between product and station records as a proportion of station observations, which can reveal over- or underestimations. $Corr$ characterizes the linear consistency between the product and reference time series, and $RMSE$ describes the overall error magnitude of the product. Detection metrics were used to describe the ability of products to identify rainfall event occurrences. Similar to hit rates, $POD$ reflects the proportion of correctly detected events (equal to 1 when all true rainfall events are detected). $FAR$ refers to the proportion of false positive events (smaller values mean fewer misidentified events). Lastly, $ETS$ characterizes the overall detection capability, representing the proportion of correct detection after considering the probability of random hits. This score ranges from − 1/3 to 1, with larger values indicating better detection capabilities. These metrics were calculated according to Eqs. (4)– (9):

4 $$RB=\frac{\sum_{i=1}^{n}\left ({x}_{i}-{y}_{i}\right)}{\sum_{i=1}^{n}{y}_{i}}\times 100\mathrm{ \%},$$

5 $$Corr=\frac{\sum_{i=1}^{n}\left ({x}_{i}-\overline{x }\right)\left ({y}_{i}-\overline{y }\right)}{\sqrt{\sum_{i=1}^{n}{\left ({x}_{i}-\overline{x }\right)}^{2}\sum_{i=1}^{n}{\left ({y}_{i}-\overline{y }\right)}^{2}}},$$

6 $$RMSE=\sqrt{\sum_{i=1}^{n}\frac{{ ({x}_{i}-{y}_{i})}^{2}}{n},}$$

7 $$POD=\frac{H}{H+M},$$

8 $$FAR=\frac{F}{H+F},$$

9 $$ETS=\frac{H-{H}_{e}}{H+M+F-{H}_{e}}, {H}_{e}=\frac{ (H+M) (H+F)}{H+M+F+C},$$

where $y$ represents the station observations; $x$ is the corresponding gridded estimates for station measurements; $n$ denotes the total number of records for the station; $H$ is the number of events detected by both station and satellite/reanalysis products; $M$ represents the number of events observed by station, but not products;$F$ is the number of events observed by products, but not station; $C$ is the number of events not observed by either station or products; and ${H}_{e}$ denotes the number of events correctly detected by the products due to randomness. To eliminate the undue influence of very light “drizzle”, a value of 1 mm/d^104^ was used instead of 0 mm/d to identify rainfall event occurrence.

Notably, when station observations were missing or invalid on a given day, the daily records for the station and corresponding product grids were excluded from the metric calculation. A total of 26 ground-based observations were missing or invalid. These records appeared at six stations (three stations with one, and the other three stations with four, five, and 14 missing records, respectively). Accordingly, the number of invalid observations at these stations was small compared to the length of the evaluation period, and did not have a significant impact on the evaluation results.

### Precipitation intensity classification standard

In addition to overall performance, evaluation was also conducted for different precipitation intensities. Therefore, daily precipitation was divided into seven levels according to the standard of the WMO^105^: < 1 mm/d, 1–2 mm/d, 2–5 mm/d, 5–10 mm/d, 10–20 mm/d, 20–50 mm/d, and ≥ 50 mm/d, referring to no/tiny rain, light rain, low moderate rain, high moderate rain, low heavy rain, high heavy rain, and violent rain, respectively.

### Error decomposition

To identify error sources, estimation errors were decomposed for each precipitation product using the Willmott decomposition technique^106^. Here, total error was split into systematic and random errors, where the former refers to the error that can be fitted with a linear function, whereas the latter is the error caused by small random fluctuations in the relevant factors during production^107^. All corresponding values were calculated according to Eqs. (10)– (13):

10 $$\frac{1}{n}\left (\sum_{i=1}^{n}{\left ({x}_{i}-{y}_{i}\right)}^{2}\right)=\frac{1}{n}\left (\sum_{i=1}^{n}{\left ({{x}^{*}}_{i}-{y}_{i}\right)}^{2}\right)+\frac{1}{n}\left (\sum_{i=1}^{n}{\left ({x}_{i}-{{x}^{*}}_{i}\right)}^{2}\right),$$

11 $${E}_{s}=\frac{{\sum }_{i=1}^{n}{\left ({{x}^{*}}_{i}-{y}_{i}\right)}^{2}}{\sum_{i=1}^{n}{\left ({x}_{i}-{y}_{i}\right)}^{2}},$$

12 $${E}_{r}=\frac{{\sum }_{i=1}^{n}{\left ({x}_{i}-{{x}^{*}}_{i}\right)}^{2}}{\sum_{i=1}^{n}{\left ({x}_{i}-{y}_{i}\right)}^{2}},$$

13 $${{x}^{*}}_{i}=a\times {y}_{i}+b,$$

where $x$, $y$ and $n$ are the same as Eqs. (4)– (6); ${E}_{s}$ and ${E}_{r}$ represent the systematic and random errors, respectively; while $a$ and $b$ denote the slope and intercept of the additive error model, respectively^108^.

### Spatial statistical analysis

To better characterize the product ability to capture precipitation spatial patterns, spatial spearman correlation coefficient ($\rho$) was used to assess the consistency of spatial distributions from the products and stations. This coefficient is a rank correlation method which can provide a better measure of spatial trend similarities. It was calculated for each day, month, and year to represent how products performed across different temporal scales. The coefficient can be obtained according to Eq. (14):

14 $$\rho =1-\frac{6\sum {\left ({x}_{i}-{y}_{i}\right)}^{2}}{{N}^{3}-N},$$

where ${x}_{i}$ and ${y}_{i}$ represent the corresponding sequence number of the product and reference records after sorting by precipitation amount from smallest to largest, respectively; and $N$ is the number of station observations.

## Results

### Evaluation for precipitation changes

#### Precipitation temporal changes

**Figure 2:**
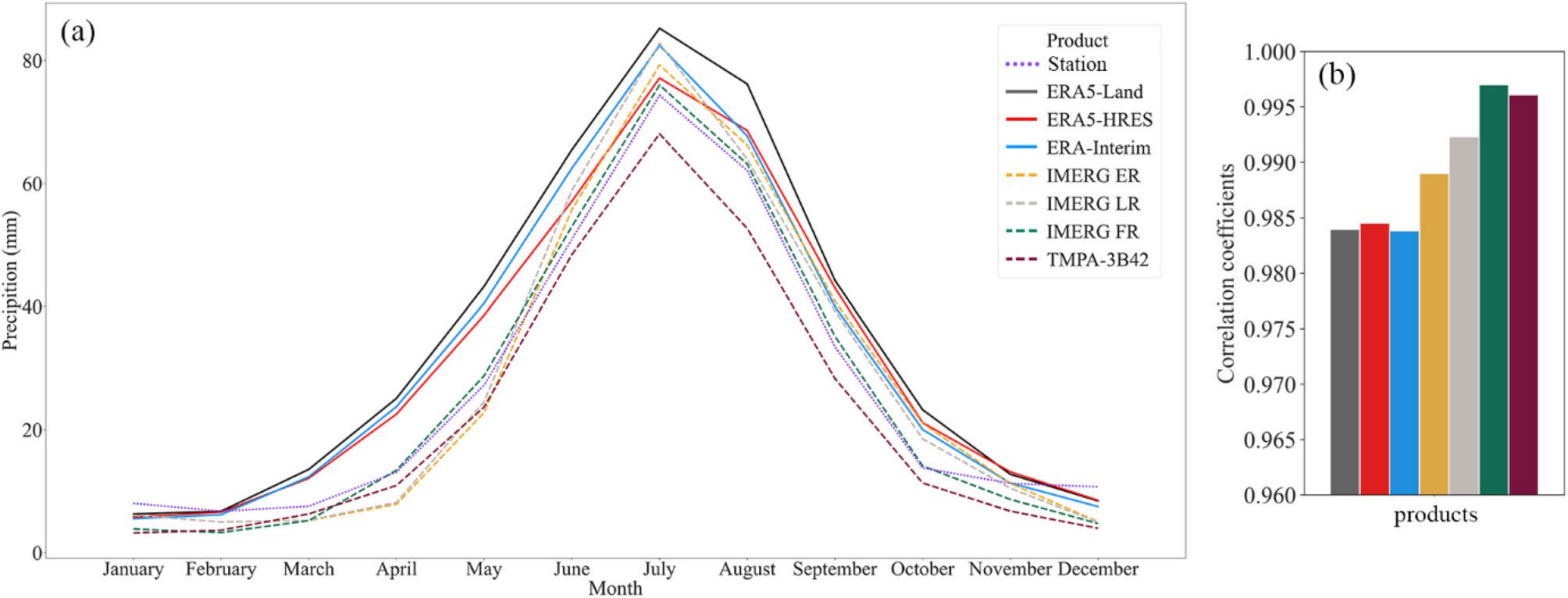
Ability of each product to describe seasonal precipitation cycles: (**a**) monthly cycles of products and ground-based stations, and (**b**) Pearson correlation coefficients between the product and station cycle series.

Figure 2 shows the seasonal cycles from the products and stations, as well as the Pearson correlation coefficients between the corresponding cycle series. These cycle series were obtained by arithmetically averaging the multi-year mean precipitation for each month from all stations or corresponding gridded estimates over the Mongolian Plateau. Monthly precipitation cycles observed by the stations followed an inverted U-shape, with a July maximum (Fig. 2a). Notably, precipitation changes were insignificant towards the beginning (January–March) and end (October–December) of the year; whereas the respective rises and drops were sharp during March–July and July–October. All products broadly captured this seasonal cycle, with high correlation coefficients between their cycle series and observations (Fig. 2b). However, they were less accurate when describing precipitation magnitude and times of significant precipitation changes. For example, the three reanalysis products overestimated monthly precipitation from March to October, as well as showed earlier precipitation rise and more significant drop at the end of the year. In comparison, the three IMERG products (particularly IMERG FR) provided better estimates from April to October, and had higher correlation coefficients. However, they were still poor at reproducing the end-of-year precipitation changes. Although persistently underestimating precipitation, TMPA-3B42 provided one of the best seasonal pattern characterizations among all products, with a slightly lower correlation coefficient than IMERG FR.

**Figure 3:**
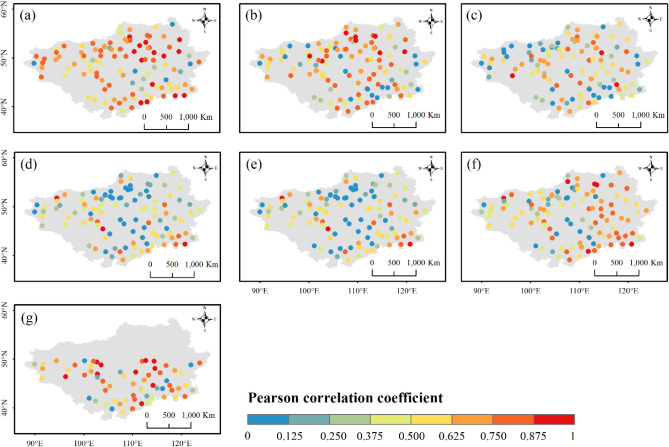
Pearson correlation coefficients between precipitation interannual variability from stations and (**a**) ERA5-Land, (**b**) ERA5-HRES, (**c**) ERA-Interim, (**d**) IMERG ER, (**e**) IMERG LR, (**f**) IMERG FR, and (**g**) TMPA-3B42. For subplot (**g**), only stations within the spatial coverage of TMPA-3B42 (50° N–50° S) were plotted. The figure was created using ArcGIS 10.7 (https://www.esri.com/en-us/arcgis/about-arcgis/overview)^70^.

**Figure 4:**
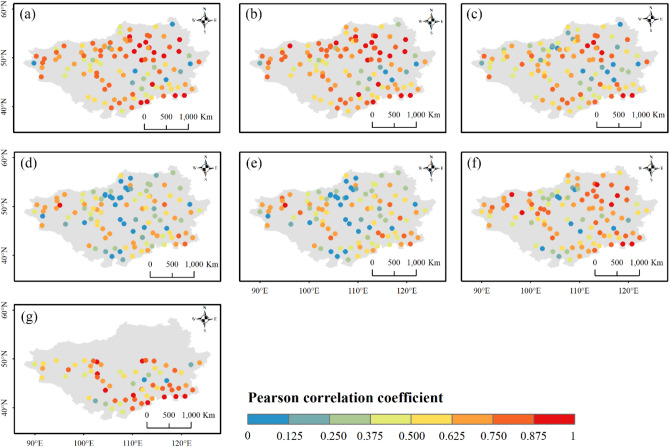
Pearson correlation coefficients between the precipitation decadal variability of stations and (**a**) ERA5-Land, (**b**) ERA5-HRES, (**c**) ERA-Interim, (**d**) IMERG ER, (**e**) IMERG LR, (**f**) IMERG FR, and (**g**) TMPA-3B42. For subplot (**g**), only stations within the spatial coverage of TMPA-3B42 (50° N–50° S) were plotted. The figure was created using ArcGIS 10.7 (https://www.esri.com/en-us/arcgis/about-arcgis/overview)^70^.

Figures 3 and 4 present the performance of the evaluated products for describing interannual and decadal variability, respectively. IMERG ER, and IMERG LR failed to capture interannual variability of precipitation, with correlation coefficients below 0.59 (coefficient value corresponding to a significance of 99%) at most stations (Fig. 3). The interannual variability of IMERG FR and TMPA-3B42 showed better agreement with observations, likely due to corrections by ground-based measurements. For the reanalysis products, ERA-Interim performed comparably to IMERG FR, while the two ERA5 products exhibited the best ability to capture interannual variability, as ERA5-HRES and ERA5-Land had correlation coefficients exceeding 0.59 at 72.7% and 65.5% of the stations, respectively. Similar results were found for decadal variability. Correlation coefficients for IMERG ER and IMERG LR remained low, while ERA5-HRES, ERA5-Land, and IMERG FR were better correlated with station data. Moreover, the products generally characterized decadal variability slightly better than interannual variability, with more stations showing correlation coefficients > 0.59.

#### Precipitation spatial patterns

**Figure 5:**
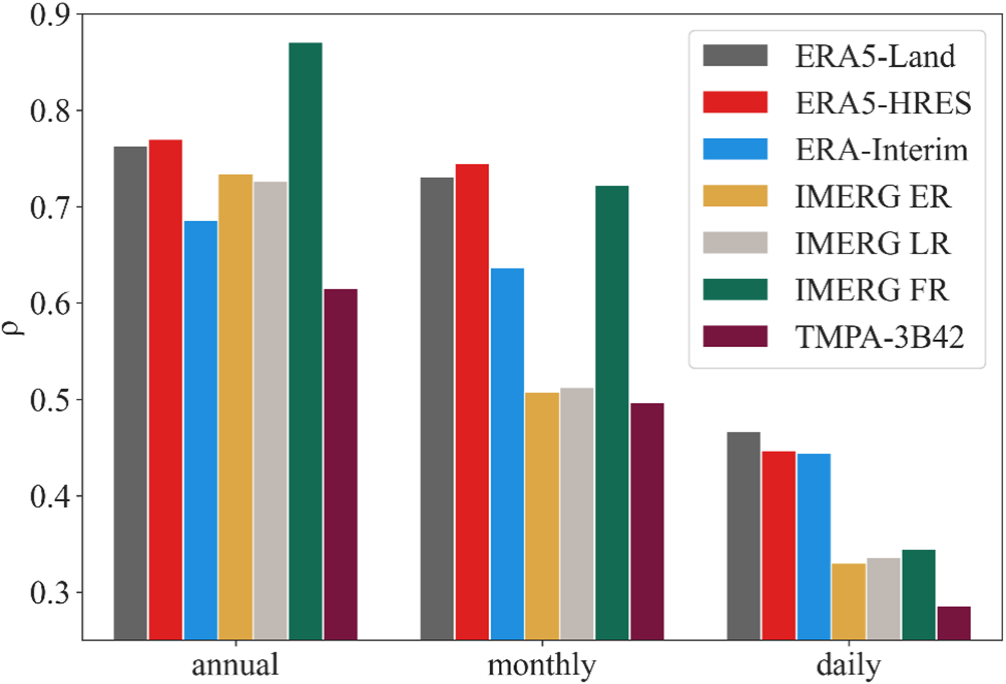
Average spatial Spearman correlation coefficients for each product at different temporal scales.

Figure 5 shows the average spatial correlation coefficients at different temporal scales. At daily scale, the reanalysis products (especially ERA5-Land) were superior to satellite products, as evidenced by the significantly higher ρ of the three reanalysis datasets. Similar results were also found in different seasons (Fig. S1), but the disparity between the two types of products was more significant during winter than summer, which may be related to the deficiency of satellites at detecting weak and solid precipitation in the winter. At monthly scale, IMERG FR showed comparable performance to the ERA5 products, probably due to GPCC corrections. Although also corrected by ground measurements, TMPA-3B42 did not perform well, which was largely due to its limited spatial coverage. For annual precipitation, spatial patterns of IMERG products were more consistent with the stations, while ERA5-HRES and ERA5-Land also exhibited relatively good ability. By contrast, the capabilities of ERA-Interim and TMPA-3B42 were weaker. Notably, ERA5-Land outperformed ERA5-HRES at daily scales, while opposite results were found at monthly and annual scales. This suggested that ERA5-Land is suitable for applications at fine spatial and temporal scales, while ERA5-HRES is more applicable to large-scale studies. All satellite and reanalysis products better described precipitation spatial patterns at coarser temporal scales, likely a result of bias offsetting.

### Daily precipitation estimation and detection

#### Overall performance

Table 2 lists the evaluation metrics for each product across the study period. Regarding estimation accuracy, all the seven products generally provided wetter estimates ($RB$ > 0). According to the $RMSE$, ERA5-HRES and ERA5-Land had the smallest errors among all products, followed by ERA-Interim and the three IMERG products. With the overall highest $RMSE$, TMPA-3B42 showed the weakest estimation ability. The evaluation for temporal linear consistency displayed similar results, with $Corr$ for each product ranking ERA5-Land > ERA5-HRES > ERA-Interim > IMERG FR > IMERG LR > IMERG ER > TMPA-3B42. Regarding the detection metrics, ERA5-Land had the highest hit rates and lowest misreport rates, resulting in high $ETS$ scores for its stronger overall detection ability. By comparison, ERA5-HRES and ERA-Interim displayed relatively weaker detection capabilities, as they missed some precipitation events (smaller $POD$). With lower $POD$ and larger $FAR$, the four satellite products exhibited poor detection capabilities, ranking IMERG FR > IMERG LR > IMERG ER > TMPA-3B42. Generally, ERA5-Land showed the best results across all metrics, followed by ERA5-HRES and ERA-Interim. IMERG products displayed a moderate ability to describe daily precipitation, with IMERG FR performing best. In contrast, TMPA-3B42 had the weakest ability to describe daily precipitation.

**Table 2:** Summary of evaluation metrics for the precipitation products for the whole study period.

Products	$RB$ (%)	$Corr$	$RMSE$ (mm/d)	$POD$	$FAR$	$ETS$
ERA5-Land	29.46	0.55	3.24	0.71	0.51	0.33
ERA5-HRES	24.97	0.50	3.17	0.67	0.52	0.31
ERA-Interim	25.73	0.49	3.44	0.62	0.52	0.28
IMERG ER	12.99	0.36	3.77	0.49	0.61	0.20
IMERG LR	13.43	0.37	3.76	0.49	0.60	0.20
IMERG FR	12.28	0.41	3.55	0.52	0.58	0.22
TMPA-3B42	0.63	0.33	3.80	0.41	0.62	0.18

#### Performance across rainy and non-rainy seasons

To compare product performances across the rainy and non-rainy seasons, metrics for the winter (DJF) and summer (JJA) were compared. As shown in Table 3, all the products experienced a decrease in overestimation magnitudes, or shift from over- to underestimation from summer to winter, suggesting that dry biases were more frequent in winter. In addition, all product estimates showed stronger agreement with stations in the summer, although $RMSE$, which is notably sensitive to precipitation amount, was also higher during this season. Similarly, the results of detection metrics indicated that the products had weaker detection capabilities in winter, showing significantly lower hit and higher misreport rates. Compared to reanalysis products, the detection ability of the four satellite products deteriorated more substantially in the winter ($FAR$ increased by > 57.7%, while $POD$ and $ETS$ decreased > 74.0% and 82.6%, respectively), which somewhat demonstrated the poor performance of the satellite products to represent winter solid precipitation.

**Table 3:** Summary of evaluation metrics for the precipitation products in the winter and summer.

Seasons	Products	$RB$ (%)	$Corr$	$RMSE$ (mm/d)	$POD$	$FAR$	$ETS$
Winter	ERA5-Land	2.35	0.48	2.10	0.51	0.54	0.28
	ERA5-HRES	1.08	0.48	2.12	0.48	0.59	0.24
	ERA-Interim	− 1.78	0.47	2.14	0.45	0.56	0.24
	IMERG ER	− 3.39	0.43	2.40	0.15	0.82	0.02
	IMERG LR	− 3.46	0.43	2.40	0.15	0.81	0.03
	IMERG FR	− 11.45	0.44	2.27	0.15	0.81	0.04
	TMPA-3B42	− 0.78	0.42	2.58	0.13	0.85	0.02
Summer	ERA5-Land	11.20	0.63	5.95	0.74	0.47	0.30
	ERA5-HRES	7.21	0.63	6.13	0.70	0.46	0.28
	ERA-Interim	10.56	0.63	6.27	0.67	0.47	0.25
	IMERG ER	9.60	0.62	6.76	0.62	0.52	0.22
	IMERG LR	11.02	0.62	6.87	0.62	0.51	0.23
	IMERG FR	3.61	0.62	6.44	0.62	0.50	0.23
	TMPA-3B42	7.95	0.60	7.07	0.50	0.52	0.19

#### Performance across precipitation intensities

**Figure 6:**
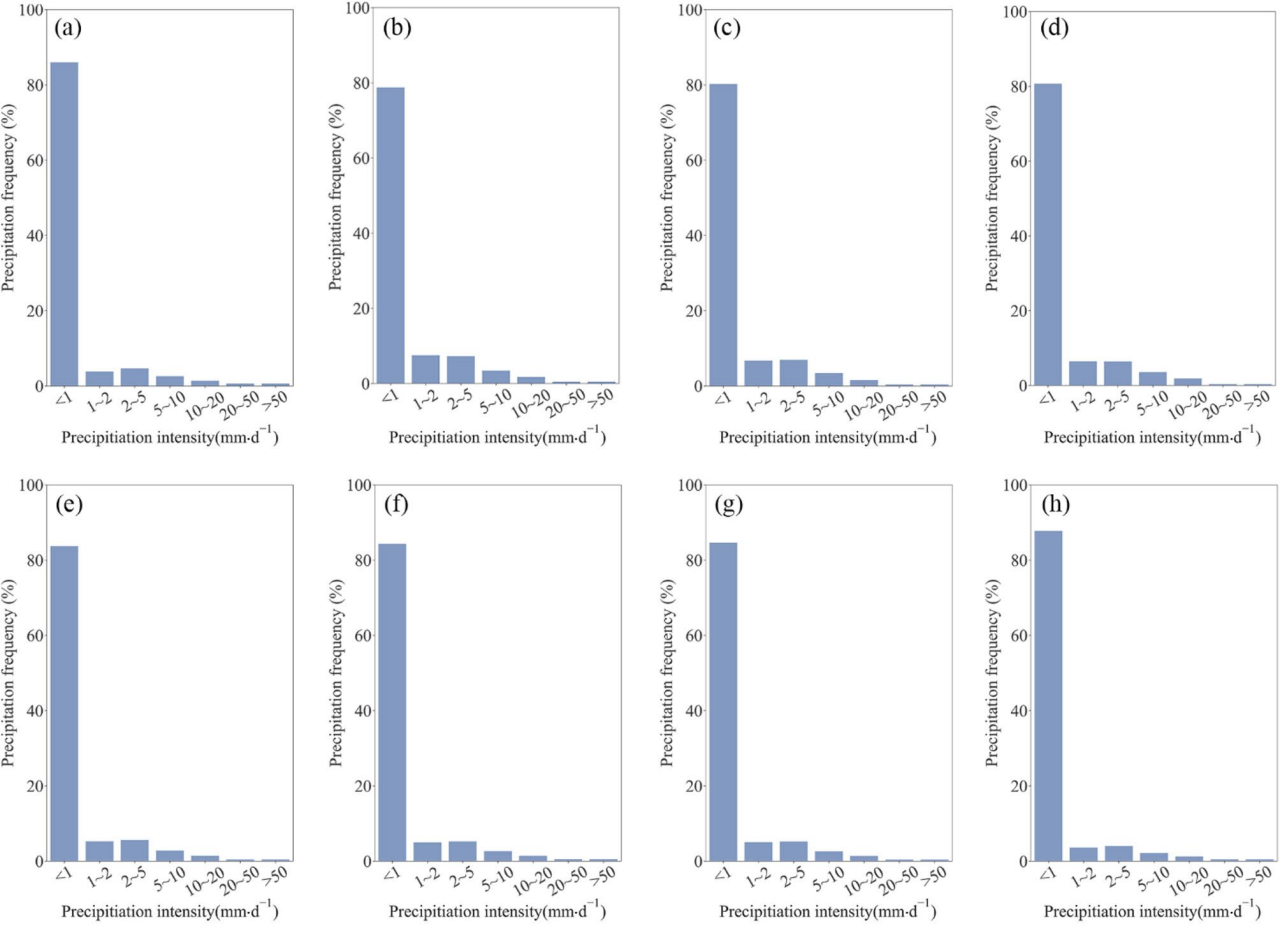
Frequency of different precipitation intensities obtained by (**a**) ground-based stations, (**b**) ERA5-Land, (**c**) ERA5-HRES, (**d**) ERA-Interim, (**e**) IMERG ER, (**f**) IMERG LR, (**g**) IMERG FR, and (**h**) TMPA-3B42.

Figure 6 exhibits the precipitation frequencies at different intensity levels obtained by the station and products. No/tiny rain was the most common on the Mongolian Plateau over the study period (frequency ~ 86%; Fig. 6a). Light, low moderate and high moderate rains occurred with moderate frequency of 3.9%, 4.7% and 2.7% respectively; while heavy and violent rain were relatively rare. The evaluated products all successfully captured this frequency pattern, but estimates were still biased. For example, the three reanalysis products significantly underestimated the frequency of no/tiny rain and overestimated that of light and moderate rains. The three IMERG products showed similar deficiencies to reanalysis data, but their frequency distributions were slightly more consistent with the reference. By contrast, TMPA-3B42 provided a more accurate description of precipitation frequency among the products.

**Figure 7:**
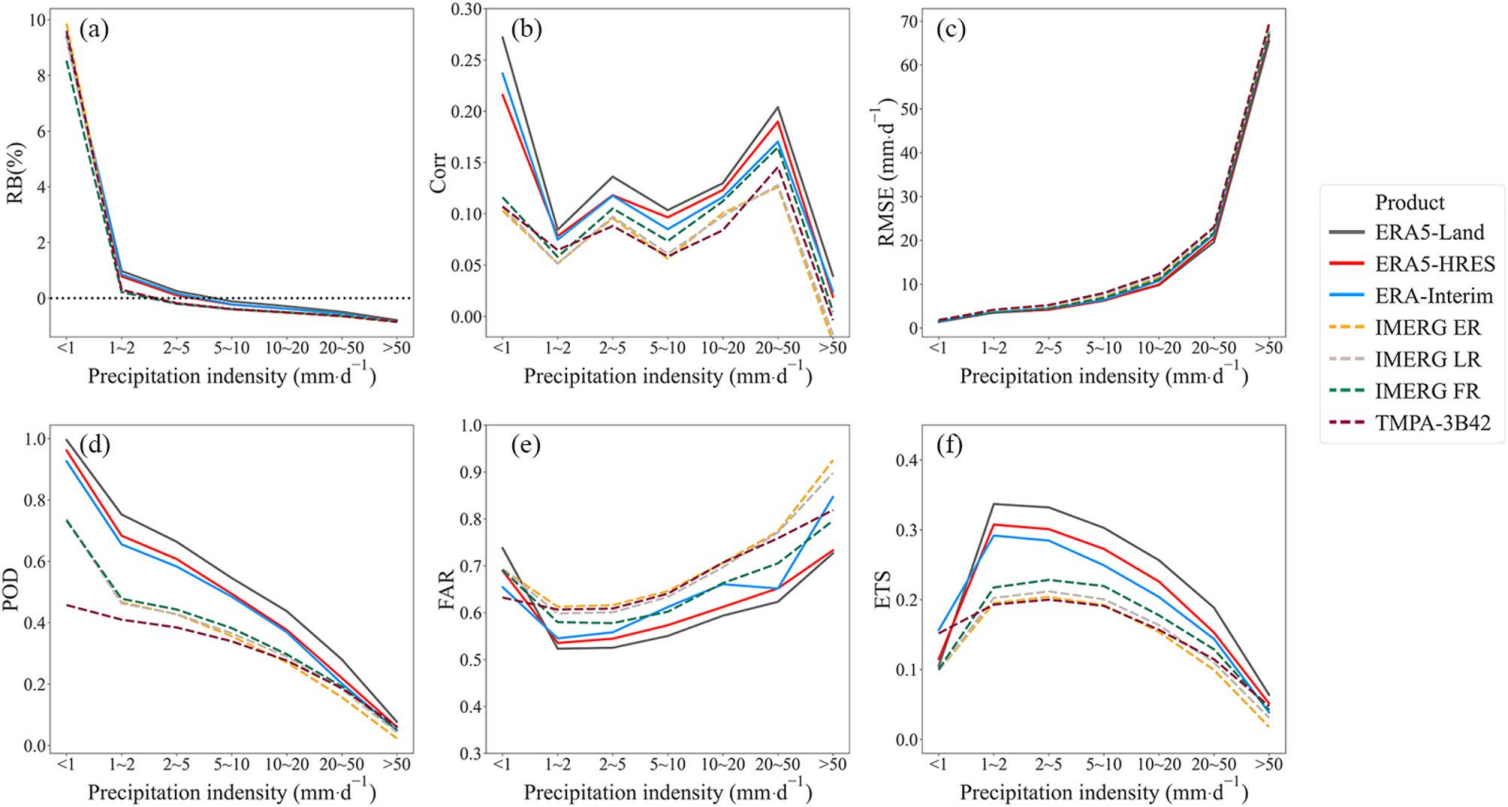
Changes in (**a**) $RB$, (**b**) $Corr$, (**c**) $RMSE$, (**d**) $POD$, (**e**) $FAR$, and (**f**) $ETS$ across different precipitation intensity levels.

Figure 7 presents the product performance under different precipitation intensities. It can be seen that the metrics for all products changed similarly with precipitation intensity. As precipitation intensity increased, $RB$ generally shifted from positive to negative, with conversion points occurring between 2 and 10 mm/d. This indicated an overestimation of weak, as well as an underestimation of high-intensity precipitation for the evaluated products. $RMSE$ displayed a sharp increasing trend, while $Corr$ generally decreased, with dual peaks occurring at low moderate and high heavy levels. For the detection metrics, the lower bounds of each range were used as the thresholds for metric calculation. Overall, $POD$ and $FAR$ displayed a monotonic decreasing and increasing trend respectively, which indicated deficiencies of the evaluated products in capturing actual heavy precipitation events. Changes in $ETS$ showed an inverted U-shape, with optimal values achieved at 1–10 mm/d. Considering the accuracy and detection metrics together, the products performed best at describing light and moderate rains.

#### Annual trends of performance

**Figure 8:**
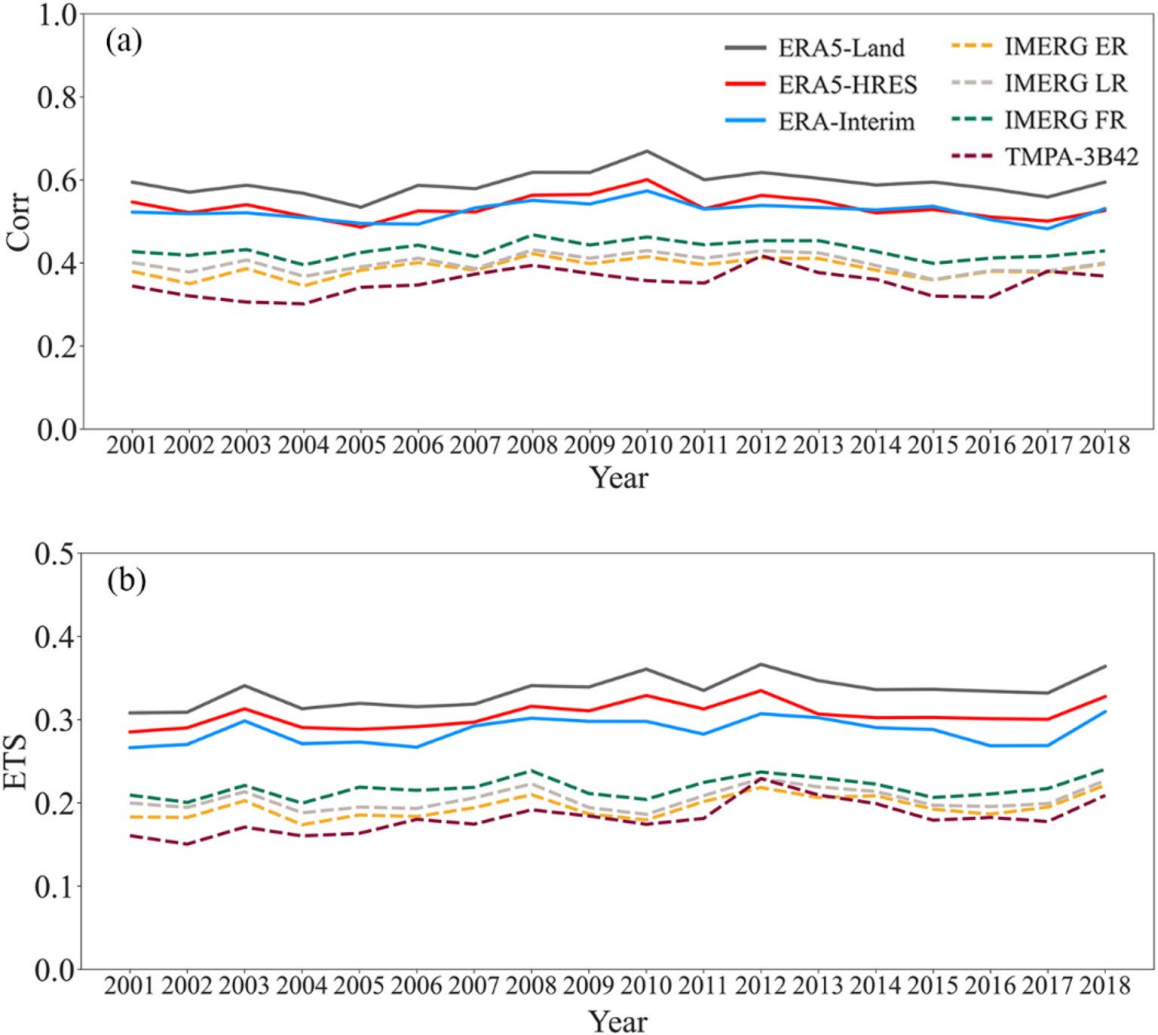
Annual (**a**) $Corr$ and (**b**) $ETS$ for different precipitation products from 2001 to 2018.

For precipitation products with long temporal coverage, understanding the stability of their performances is critical to their practical use. Therefore, $Corr$ and $ETS$ were selected as representative accuracy and detection metrics, respectively, to analyze the annual change of product performance. Figure 8 shows the annual changes of the two metrics, and Table 4 presents the change trends and significance following linear regressions with time. The estimation and detection capabilities of all products improved slightly over the study period, although the improvement is not significant except for the detection ability of TMPA-3B42 (F-test, p < 0.05). Overall, the performances of the evaluated products were stable between 2001 and 2018, demonstrating their effectiveness for long-term applications.

**Table 4:** Linear regression trends and corresponding significance of $Corr$ and $ETS$ for precipitation products from 2001 to 2018 (*p < 0.05).

Products	Trends of $Corr$	Trends of $ETS$
ERA5-Land	0.0008	0.0021
ERA5-HRES	0.0003	0.0010
ERA-Interim	0.0003	0.0010
IMERG ER	0.0010	0.0011
IMERG LR	0.0008	0.0008
IMERG FR	0.0008	0.0010
TMPA-3B42	0.0020	0.0024*

### Spatial characteristics of estimation and detection ability

#### Metric spatial variation

**Figure 9:**
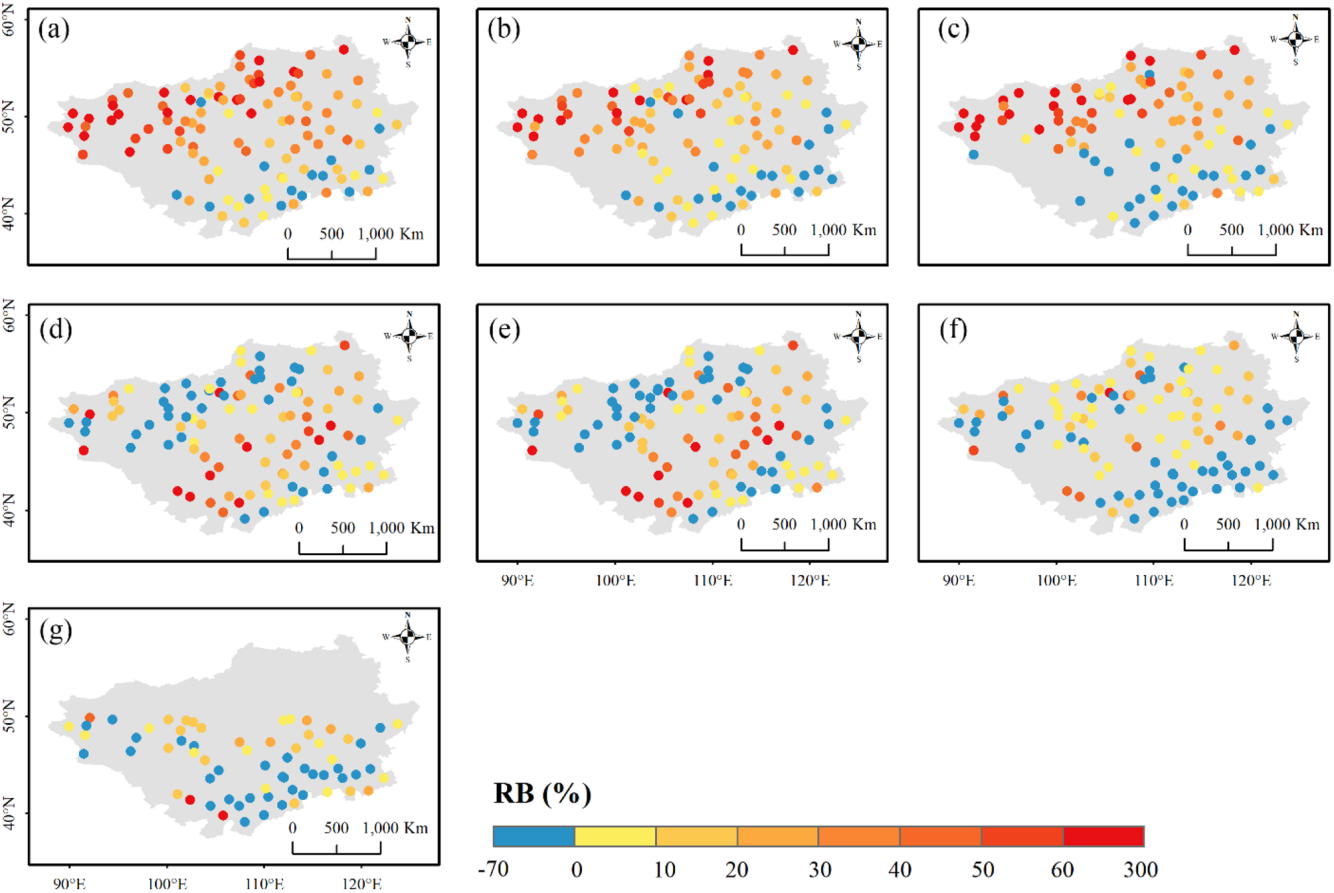
Spatial distribution of $RB$ for (**a**) ERA5-Land, (**b**) ERA5-HRES, (**c**) ERA-Interim, (**d**) IMERG ER, (**e**) IMERG LR, (**f**) IMERG FR, and (**g**) TMPA-3B42. For subplot (**g**), only stations within the spatial coverage of TMPA-3B42 (50° N–50° S) were plotted. The figure was created using ArcGIS 10.7 (https://www.esri.com/en-us/arcgis/about-arcgis/overview)^70^.

**Figure 10:**
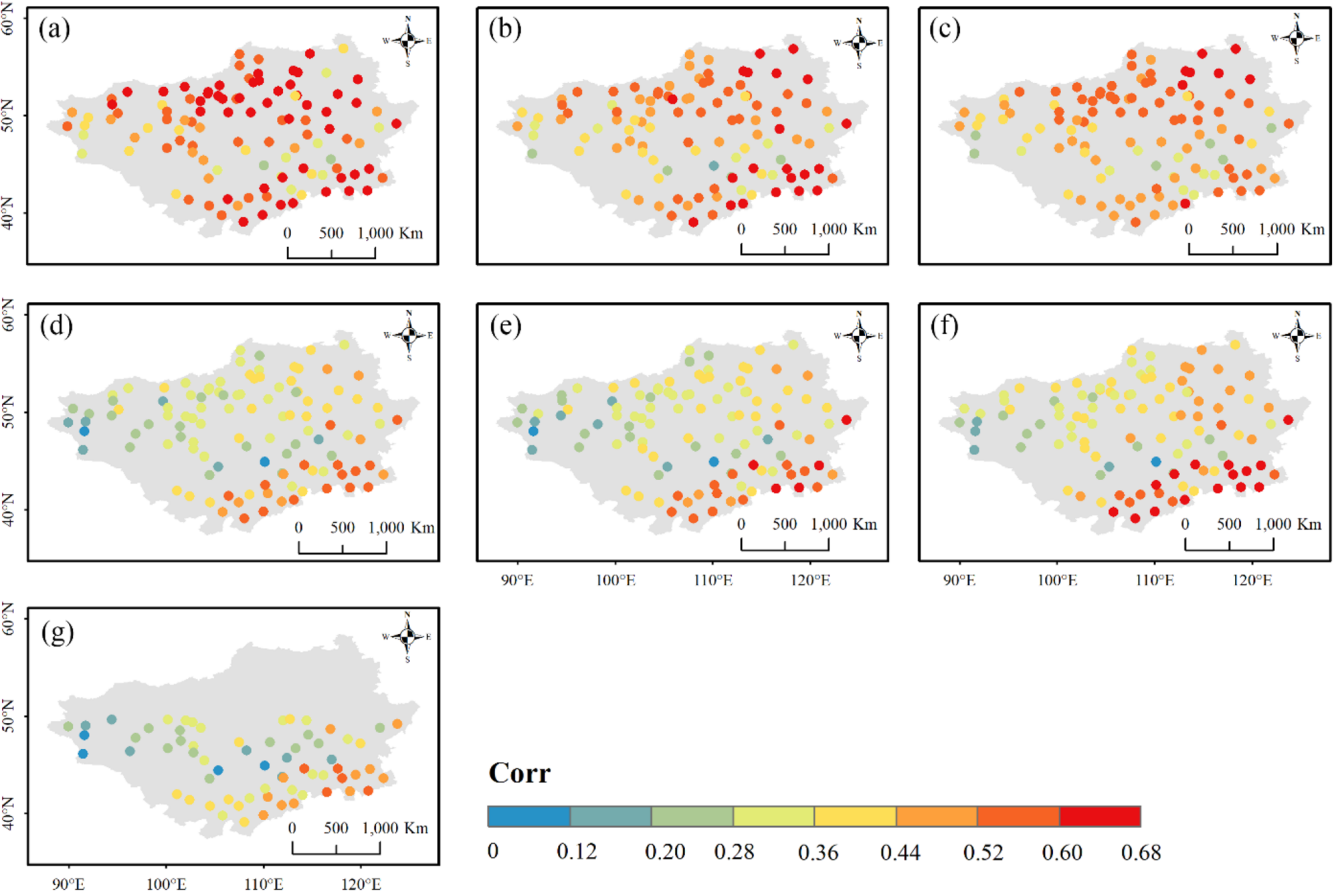
Spatial distribution of $Corr$ for (**a**) ERA5-Land, (**b**) ERA5-HRES, (**c**) ERA-Interim, (**d**) IMERG ER, (**e**) IMERG LR, (**f**) IMERG FR, and (**g**) TMPA-3B42. For subplot (**g**), only stations within the spatial coverage of TMPA-3B42 (50° N–50° S) were plotted. The figure was created using ArcGIS 10.7 (https://www.esri.com/en-us/arcgis/about-arcgis/overview)^70^.

**Figure 11:**
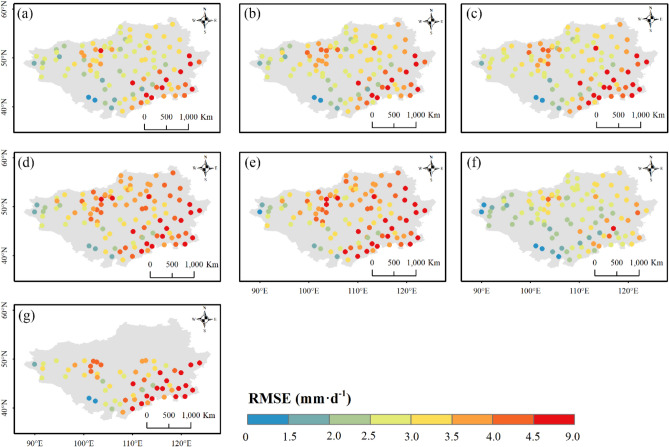
Spatial distribution of $RMSE$ for (**a**) ERA5-Land, (**b**) ERA5-HRES, (**c**) ERA-Interim, (**d**) IMERG ER, (**e**) IMERG LR, (**f**) IMERG FR, and (**g**) TMPA-3B42. For subplot (**g**), only stations within the spatial coverage of TMPA-3B42 (50° N–50° S) were plotted. The figure was created using ArcGIS 10.7 (https://www.esri.com/en-us/arcgis/about-arcgis/overview)^70^.

Apart from the overall performance at regional scales, the product ability was also assessed across different parts of the Mongolian Plateau. Figures 9, 10 and 11 presents the spatial distributions of accuracy metrics for the seven products. The three reanalysis products generally showed underestimations along the southern edge of the Plateau, with a gradual transition to overestimation from south to north (Fig. 9). Their highest relative wet biases were all found along the northern edge. Compared to ERA5-HRES, the overestimation (underestimation) of ERA5-Land (ERA-Interim) was more significant. The spatial patterns of $RB$ for the three IMERG products were similar, with two strips of dry bias zones in the south and north of the Plateau, and wet bias zones primarily in central Mongolia. Possibly benefiting from the GPCC calibration, IMERG FR avoided some obvious overestimation in the south for the two near real-time products. For TMPA-3B42, only performances to the south of 50° N could be investigated due to its limited spatial coverage. Within the effective spatial range, TMPA-3B42 also displayed underestimation at the south-eastern edge, and overestimated precipitation towards the center. Considering the temporal linear agreement, $Corr$ values of the three reanalysis products were higher at the northern and southern edges, and lower in the west and center (Fig. 10). By contrast, $Corr$ of the four satellite products showed spatial trends of decreasing from southeast to northwest. In addition, $RMSE$ scores for all products were relatively high at the southeastern edge and north-central area, mainly caused by the higher precipitation levels in these regions (Fig. S2).

**Figure 12:**
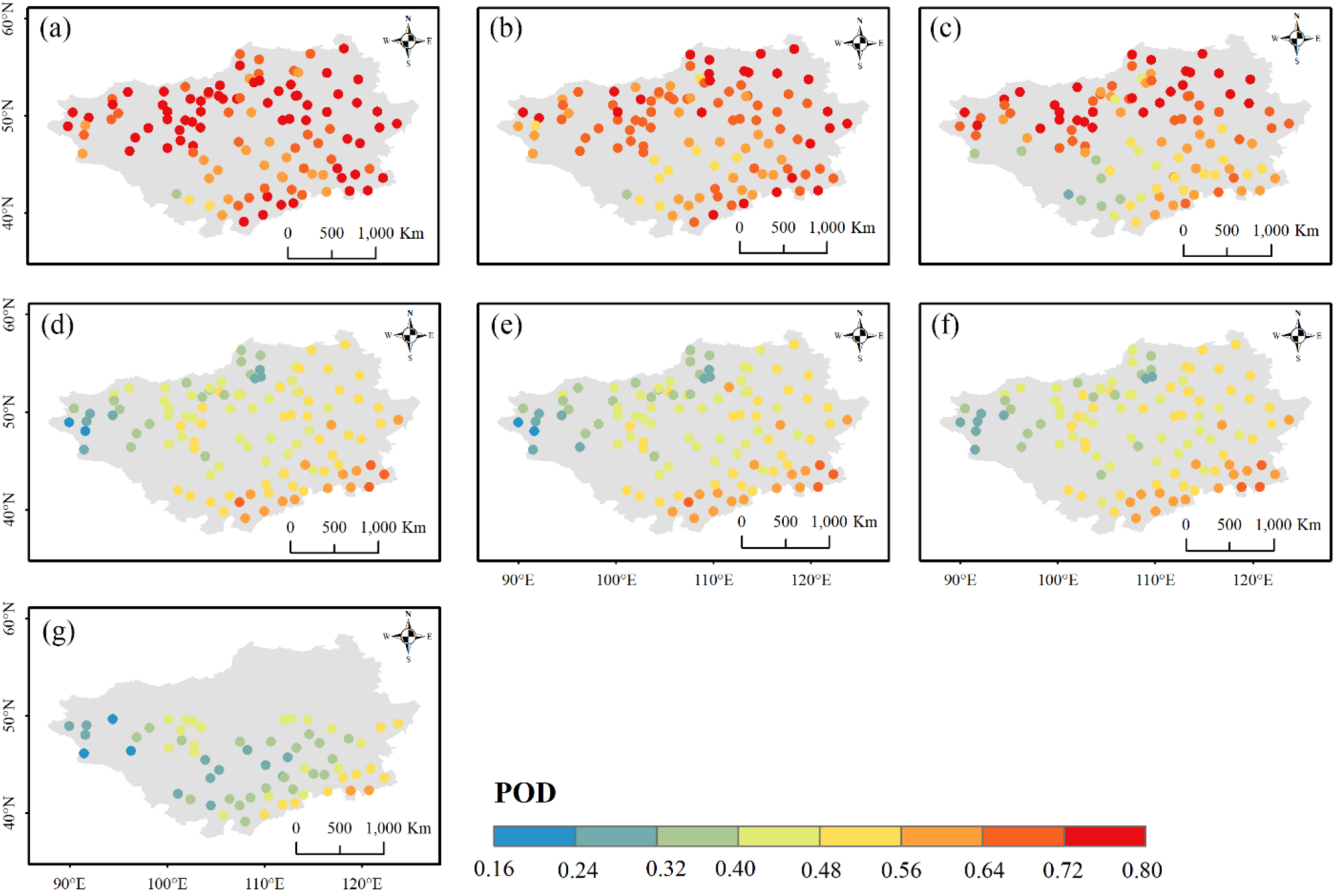
Spatial distribution of $POD$ for (**a**) ERA5-Land, (**b**) ERA5-HRES, (**c**) ERA-Interim, (**d**) IMERG ER, (**e**) IMERG LR, (**f**) IMERG FR, and (**g**) TMPA-3B42. For subplot (**g**), only stations within the spatial coverage of TMPA-3B42 (50° N–50° S) were plotted. The figure was created using ArcGIS 10.7 (https://www.esri.com/en-us/arcgis/about-arcgis/overview)^70^.

**Figure 13:**
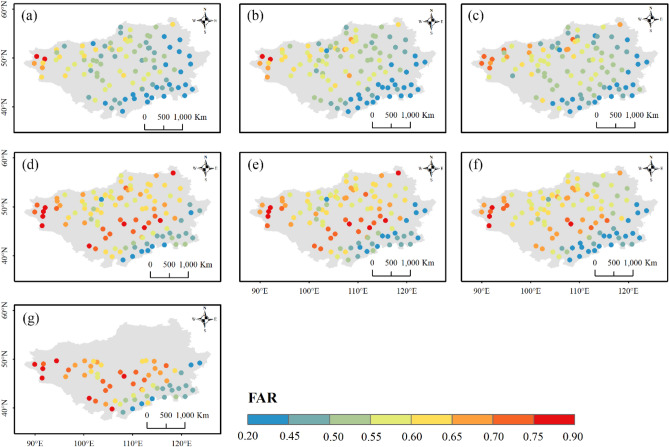
Spatial distribution of $FAR$ for (**a**) ERA5-Land, (**b**) ERA5-HRES, (**c**) ERA-Interim, (**d**) IMERG ER, (**e**) IMERG LR, (**f**) IMERG FR, and (**g**) TMPA-3B42. For subplot (**g**), only stations within the spatial coverage of TMPA-3B42 (50° N–50° S) were plotted. The figure was created using ArcGIS 10.7 (https://www.esri.com/en-us/arcgis/about-arcgis/overview)^70^.

**Figure 14:**
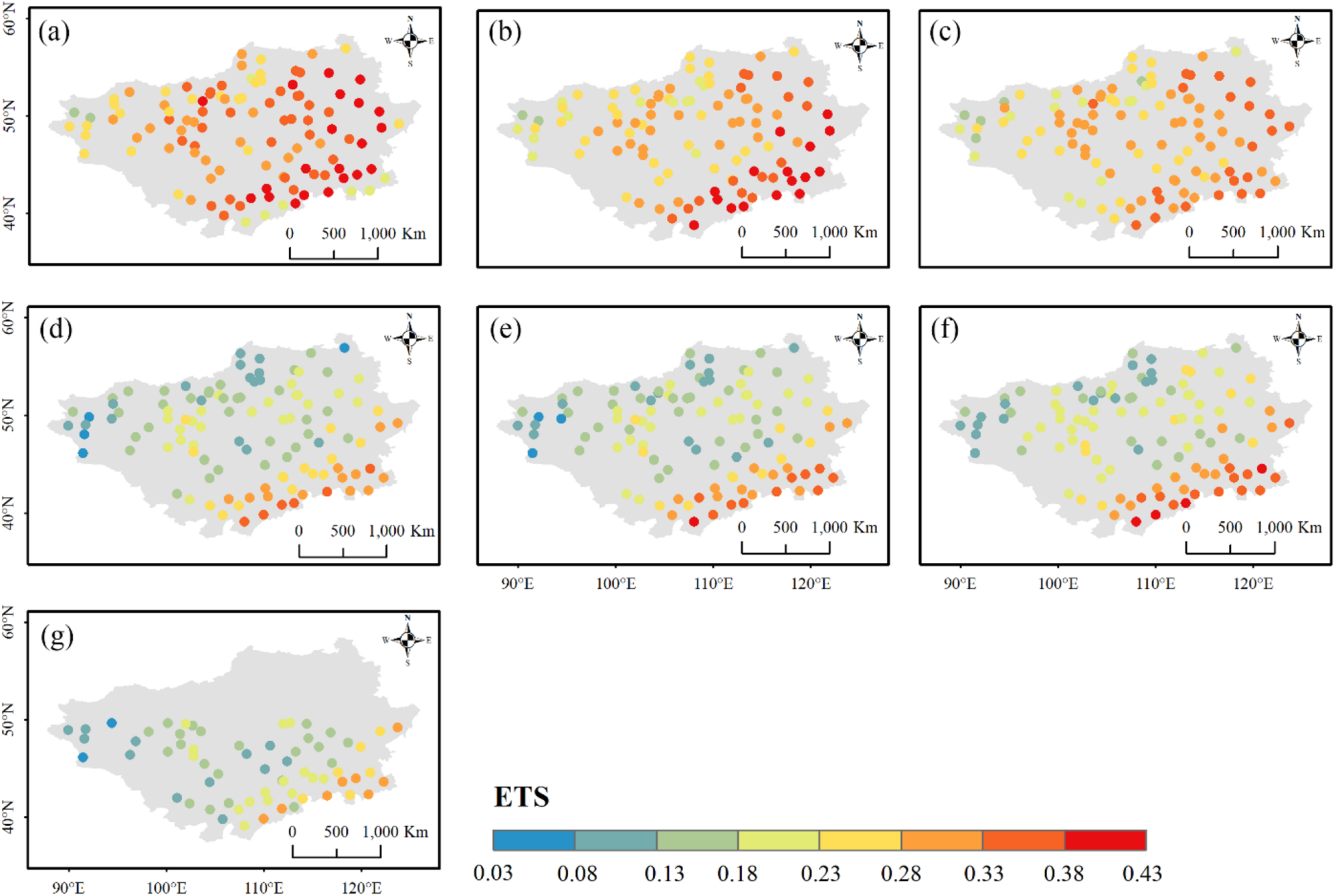
Spatial distribution of $ETS$ for (**a**) ERA5-Land, (**b**) ERA5-HRES, (**c**) ERA-Interim, (**d**) IMERG ER, (**e**) IMERG LR, (**f**) IMERG FR, and (**g**) TMPA-3B42. For subplot (**g**), only stations within the spatial coverage of TMPA-3B42 (50° N–50° S) were plotted. The figure was created using ArcGIS 10.7 (https://www.esri.com/en-us/arcgis/about-arcgis/overview)^70^.

Figures 12, 13 and 14 display the spatial distributions of the detection metrics. For $POD$, ERA5-Land, ERA5-HRES, and ERA-Interim all performed best in the north and second-best to the south of the Plateau, where the multi-year average daily precipitation intensities were mostly light to moderate (Fig. S2). In comparison, $POD$ of the four satellite products exhibited a decreasing trend from southeast to northwest. The spatial patterns of $FAR$ were generally opposite to those of $POD$, with all products achieving low values in southern Inner Mongolia and higher values to the northwestern corner of the Plateau. As a result, $ETS$ decreased gradually towards the northwest, with optimal values found to the southeast. Jointly considering the accuracy and detection metrics, all products showed a relatively superior ability to describe daily precipitation at the southern edge of the study area, while performing more poorly to the mid-west and northeast. Notably, this finding was consistent with the evaluation results under different precipitation intensities.

#### Error component decomposition

**Figure 15:**
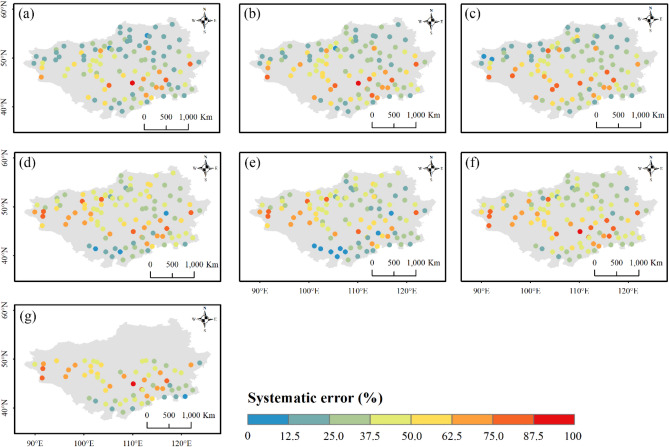
Spatial distribution of systematic errors for (**a**) ERA5-Land, (**b**) ERA5-HRES, (**c**) ERA-Interim, (**d**) IMERG ER, (**e**) IMERG LR, (**f**) IMERG FR, and (**g**) TMPA-3B42 from 2001 to 2018. For subplot (**g**), only stations within the spatial coverage of TMPA-3B42 (50° N–50° S) were plotted. The figure was created using ArcGIS 10.7 (https://www.esri.com/en-us/arcgis/about-arcgis/overview)^70^.

As random error is inversely proportional to systematic error, the systematic error results are presented here for illustration (Fig. 15), and the random error results are placed in the supplementary material (Fig. S3). ERA5-HRES, ERA5-Land and ERA-Interim had an overall systematic error smaller than the random error, with region-wide averages of 36.8%, 34.9%, and 40.1%, respectively. Compared to ERA-Interim, the two ERA5 products produced smaller systematic errors, demonstrating the improvement of ERA5 reanalysis models. Overall, the four satellite products showed slightly higher systematic error magnitudes than the reanalysis products, with regional average errors for IMERG ER, IMERG LR, IMERG FR, and TMPA-3B42 of 43.6%, 40.2%, 45.4%, and 46.9%, respectively. All products generally exhibited relatively larger systematic errors to the mid-west, where precipitation was low. This suggested potential improvement in satellite retrieval algorithms or reanalysis models for obtaining weak precipitation data in arid areas.

## Discussion

### Performance by topography

Considering the complex topography of the Mongolian Plateau, evaluations were also performed under different topographic complexities. Previous studies have used elevation to indicate the topographic complexity^37,51,76,109^. However, precipitation at high altitude areas with flat topographies may not significantly differ much from flat areas at lower altitudes. Therefore, referring to the approach of Amjad et al., slope was used here to represent topographic complexity^47^.

**Figure 16:**
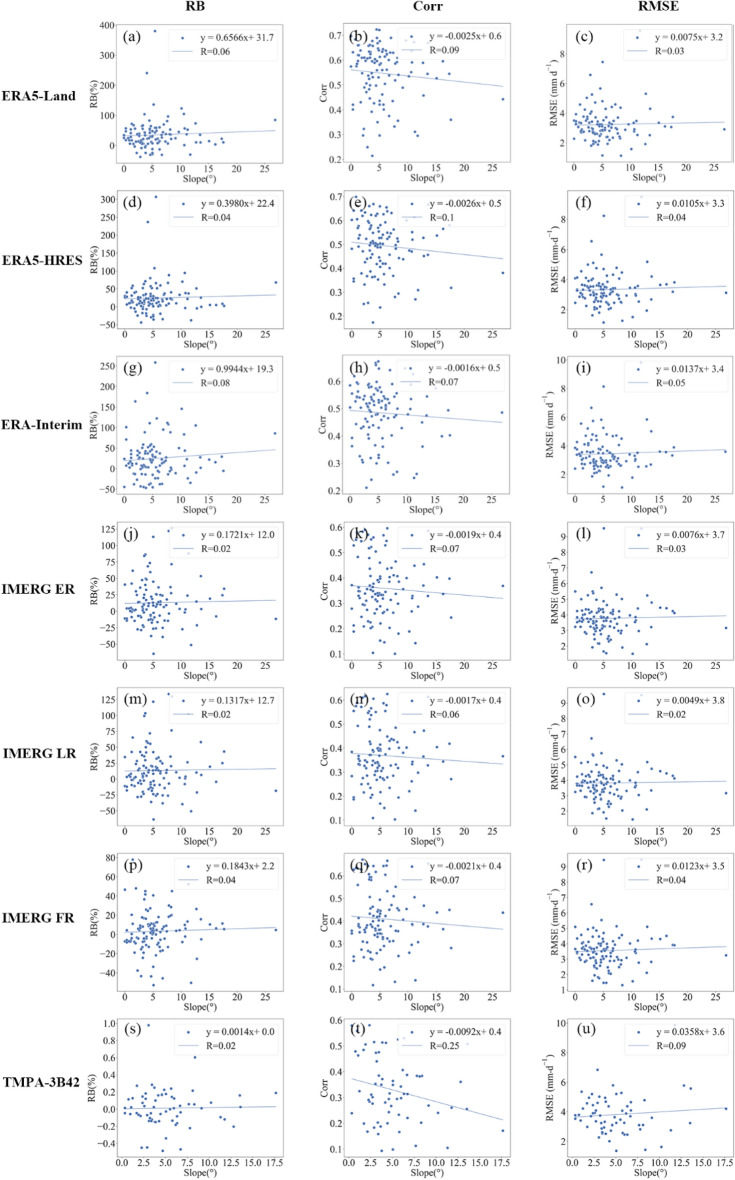
Changes in accuracy metrics with slopes. The subplots are $RB$ for (**a**) ERA5-Land, (**d**) ERA5-HRES, (**g**) ERA-Interim, (**j**) IMERG ER, (**m**) IMERG LR, (**p**) IMERG FR, (**s**) TMPA-3B42; $Corr$ for (**b**) ERA5-Land, (**e**) ERA5-HRES, (**h**) ERA-Interim, (**j**) IMERG ER, (**n**) IMERG LR, (**q**) IMERG FR, (**t**) TMPA-3B42; and $RMSE$ for (**c**) ERA5-Land, (**f**) ERA5-HRES, (**i**) ERA-Interim, (**l**) IMERG ER, (**o**) IMERG LR, (**r**) IMERG FR, (**u**) TMPA-3B42.

**Figure 17:**
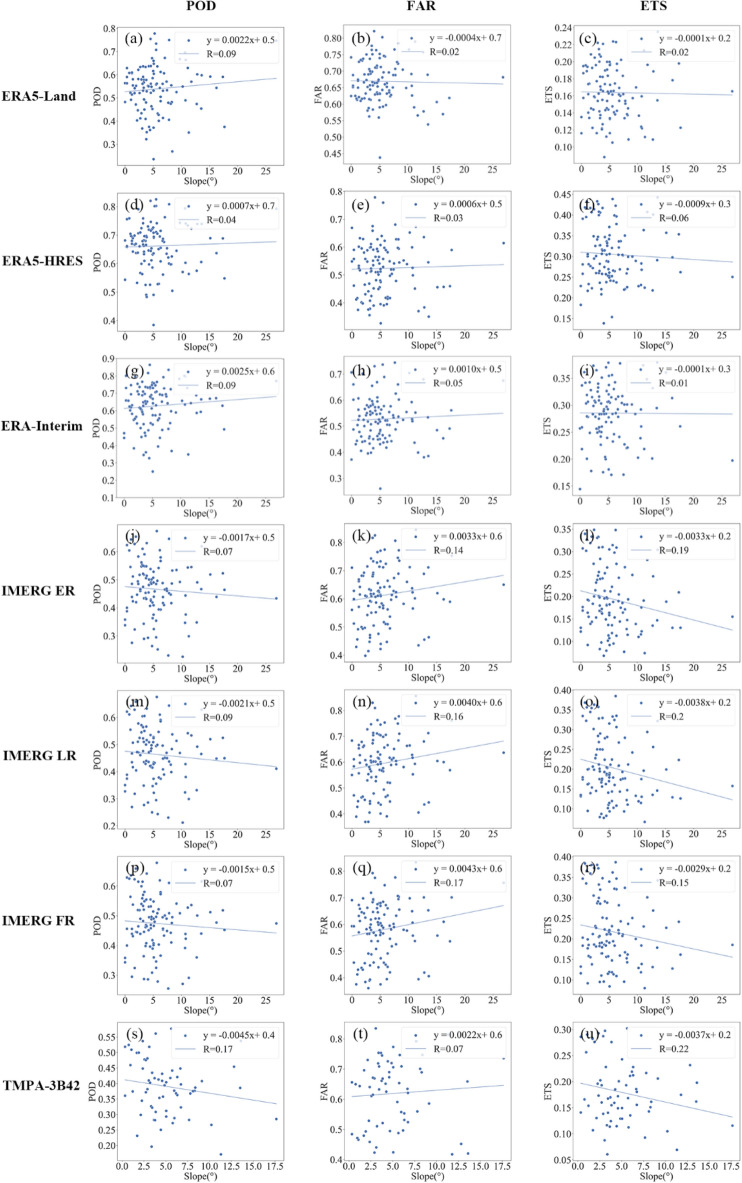
Changes in detection metrics with slopes. The subplots are $POD$ for (**a**) ERA5-Land, (**d**) ERA5-HRES, (**g**) ERA-Interim, (**j**) IMERG ER, (**m**) IMERG LR, (**p**) IMERG FR, (**s**) TMPA-3B42; $FAR$ for (**b**) ERA5-Land, (**e**) ERA5-HRES, (**h**) ERA-Interim, (**j**) IMERG ER, (**n**) IMERG LR, (**q**) IMERG FR, (**t**) TMPA-3B42; and $ETS$ for (**c**) ERA5-Land, (**f**) ERA5-HRES, (**i**) ERA-Interim, (**l**) IMERG ER, (**o**) IMERG LR, (**r**) IMERG FR, (**u**) TMPA-3B42.

Figures 16 and 17 show the changes in accuracy and detection metrics versus slope, respectively. In terms of accuracy metrics, $RB$ and $RMSE$ for all products increased with slope, albeit insignificantly (R^2^ < 0.1). Similarly, changes in $Corr$ for the evaluated products were also not significant, except for TMPA-3B42. Regarding detection metrics, slope had no apparent impact on the $POD$, $FAR$ and $ETS$ of the reanalysis data. By contrast, topographic complexity exerted a noticeable detrimental effect on the detection capabilities of IMERG and TMPA-3B42, as evidenced by the negative relationships between slope and their $ETS$ scores (R^2^ > 0.15). Nevertheless, topography had slightly different impacts on the detection process of the two generations of satellite products. In areas with more complex topography, the deficiency of IMERG was manifested as a notable increase in misdetection rate (larger $FAR$), while TMPA-3B42 fell short in terms of hit rate (lower $POD$).

The topographic effect on the detection capabilities of satellite products is possibly related to orographic precipitation which have strong spatiotemporal heterogeneities. Complex topographies can contribute to precipitation development by lifting moisture currents^110^, forming precipitation that is small in scale, short in duration, and low in intensity^111^. Limited by the observation equipment, TMPA-3B42 may often miss these types of events; whereas with a more sensitive sensor, IMERG may incorrectly identify high concentrations of water vapor that are elevated by topography but do not actually forming precipitation.

### Performance under different land cover types

Precipitation data is fundamental for drought monitoring, agricultural management, and urban planning, each of which involves different land types. Therefore, it is important to investigate product performance across a variety of land cover types. According to the CCI-LC data, 86.4% of the GSOD reference stations were located under stable land cover conditions across the study period. These stations with no land cover change were used in the analysis (Fig. S4).

**Figure 18:**
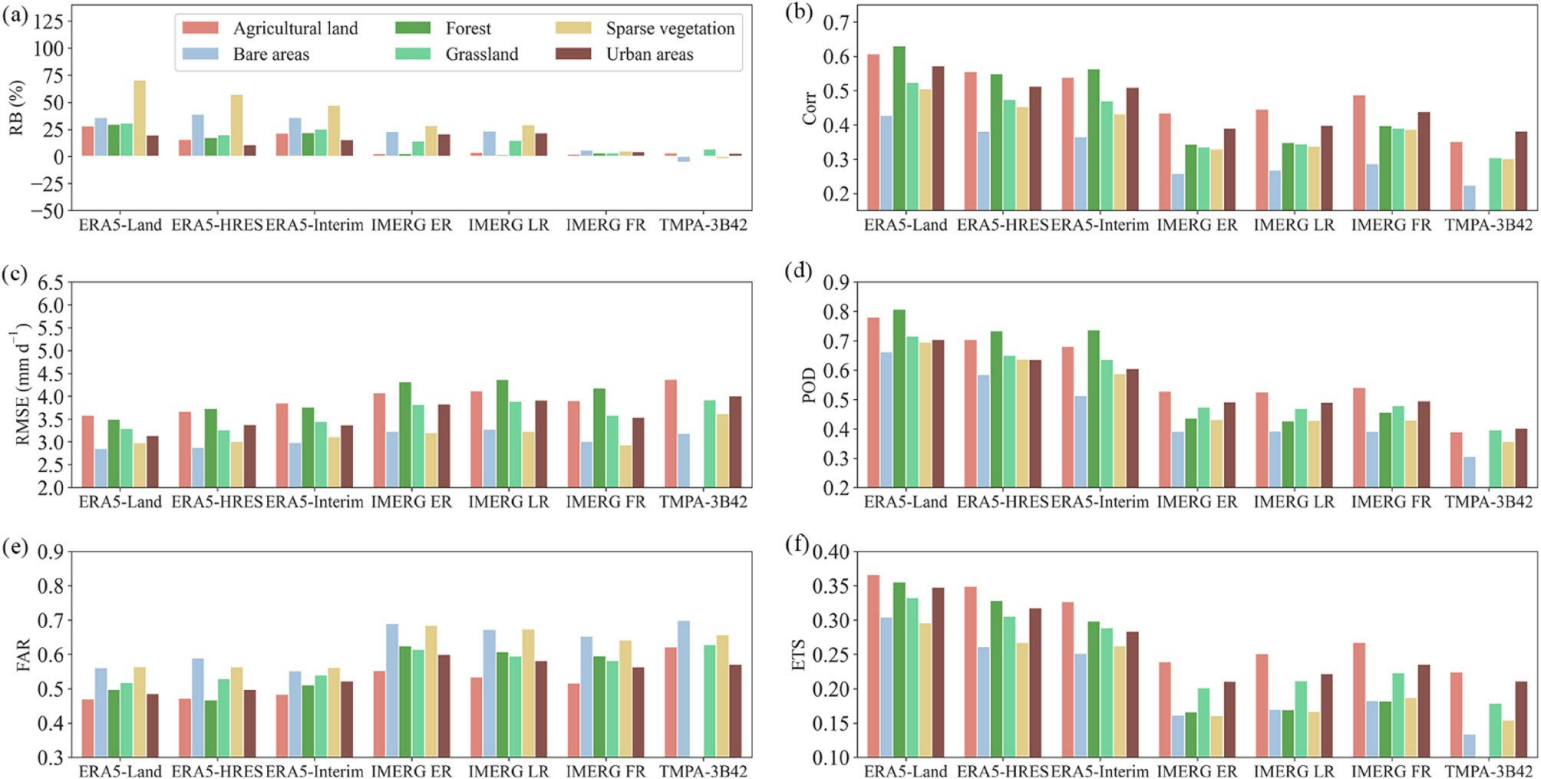
Changes in (**a**) $RB$, (**b**) $Corr$, (**c**) $RMSE$, (**d**) $POD$, (**e**) $FAR$, and (**f**) $ETS$ under different land cover types.

Figure 18 presents the changes in metrics under different land cover types. Overall, the best capabilities of ERA5-Land and ERA5-HRES were found in agricultural lands and forests, as reflected by their smaller positive $RB$ and $FAR$, as well as larger $Corr$, $POD$ and $ETS$. ERA5 performance was moderate in urban areas and grasslands, and worst in sparse vegetation and bare areas. Similar characteristics were observed for ERA-Interim and IMERG. However, the IMERG detection capability in forests was weak due to low hit and high misdetection rates. This was because the forests were mainly distributed in areas with greater slopes on the Mongolian Plateau (Fig. S4), where the detection capability of IMERG was negatively affected by topographic complexity. Due to the limitation of spatial coverage, the performance of TMPA-3B42 in forests cannot be assessed; whereas the ability differences across the other five land cover types were similar to those of IMERG.

### Strengths and weaknesses of the precipitation products

The evaluation for precipitation patterns showed that both the satellite and reanalysis products could generally capture precipitation seasonal cycle and spatial distributions. Similar results are found in mainland China^112^, the Himalayas^113^, South Asia^114^, Brazil^115^, and Europe^116^ for IMERG, as well as in eastern China^117,118^, the Tibetan Plateau^119^, the Pamir Plateau^45^, and Africa^120^ for ERA5. With direct corrections by monthly GPCC observations, IMERG FR provided more accurate monthly estimates, and performed best in capturing the seasonal cycle and annual spatial distributions. At daily scale, ERA5 products showed better capability for spatial pattern, probably attributed to their better characterizations of daily precipitation. For long-term changes, ERA5 outperformed IMERG in capturing both interannual and decadal variability, which is also noted on the Tibetan Plateau^94^.

In terms of representing daily precipitation, ERA5 and IMERG showed superiority over their predecessors, consistent with many studies worldwide^51,55,121–123^. However, further improvements were still needed for their estimation and detection capabilities. Specifically, ERA5 generally overestimated precipitation and mis-detected many non-precipitation events, which is previously reported in the Tibetan Plateau^124^, southern China^125^, Austria^46^, North America^48^, and Iran^58^. This probably stems from the imperfections in the cumulus parameterizations over steep mountain slopes^126^. By contrast, IMERG exhibited poorer estimation accuracy and weaker detection ability, especially in winter. Similar findings are noted in regions with complex topography such as Turkey^47^ and the Tibetan Plateau^41^. Limited by the observation equipment, IMERG has difficulties in detecting precipitation that is solid or weaker than 0.2 mm/h^127^, resulting in a low hit rate. However, satellites may also misidentify some dynamics land surface characteristics as precipitation signals^128^, which would cause overestimation and misdetection. Moreover, the morphing process of the IMERG algorithm is also likely to increase the occurrence of estimated precipitation^129^.

For different precipitation intensities, ERA5 and IMERG showed shortcomings of overestimating weak and underestimating high-intensity precipitation, which has been reported for eastern China^130,131^, the Himalayas^132^, North America^35^, and Central Asia^133^. The overestimation of weak precipitation can be explained by reasons for overall overestimation above. The underestimation of heavy precipitation by ERA5 may come from the deficiencies in its sub-grid convection parameterization schemes^134^; while the drier estimates from IMERG are mainly related to the weak association between some intense precipitation types and the atmospheric signatures relied on by satellite retrievals^135^.

The comparison between different products demonstrated that ERA5 generally outperformed IMERG in mountainous areas with complex topography, which has been pointed out by previous studies^54,92^. Of the two ERA5 products, ERA5-Land had better ability to estimate and detect daily precipitation, while ERA5-HRES provided better spatial pattern and long-term variability at larger temporal scales. With relatively accurate monthly estimates, IMERG FR showed the best capability in terms of seasonal cycle and annual spatial distribution. IMERG ER and IMERG LR performed most poorly among the ERA5 and IMERG products.

In addition to data quality, ERA5 and IMERG each have strengths and weaknesses concerning spatial and temporal resolution, temporal coverage, and data release latency. Compared to the 0.25° ERA5-HRES, ERA5-Land and IMERG products have a finer spatial resolution of 0.1°. With respect to temporal resolution, IMERG provides estimates at half-hourly intervals, more frequently than ERA5 (1 h). However, the two ERA5 products have decades of precipitation records (1950 to present), while the time span of IMERG covers only the last two decades (2000 to present). Besides, IMERG ER and IMERG LR are released with short delays (4 and 12 h, respectively), which shows significant advantages over ERA5-HRES, ERA5-Land and IMERG FR (delays of 5 days, 3 and 3.5 months, respectively).

Combined with the results of this study, ERA5 reanalysis products are currently more suitable than IMERG satellite data for climatic, hydrological, meteorological, and ecological applications on the Mongolian Plateau. Specifically, ERA5-HRES is applicable for long-term, large-scale research, such as interdecadal variability and trend analysis, as noted by assessments on the Tibetan Plateau^94,123^. With higher spatial resolution and better characterization of daily precipitation, ERA5-Land has great potential for topographic precipitation detection and glacio-hydrological studies, which has also been suggested by a study in mountainous areas^132^. However, IMERG products still show advantages in some cases. For example, IMERG FR has more accurate monthly records and can be applied to studies of hydrological processes in watersheds at seasonal scale. In addition, IMERG ER has short release latency as well as fine spatial and temporal resolution, providing great opportunities for weather forecasting and flood warning, although further improvements are still critical.

## Conclusions

The Mongolian Plateau is a typical arid and semi-arid region with particularly fragile ecological environment and sensitivity to climate change. Precipitation has a significant impact on the regional ecological environment and social development, which make reliable precipitation data urgently needed. Due to the sparse distribution of stations, spatially continuous global satellite and reanalysis data holds great significance for regional studies on the Mongolian Plateau. Among the various products, IMERG and ERA5 are representative of the modern satellite and reanalysis precipitation datasets, respectively. With high-quality records, fine resolutions, and wide spatiotemporal coverage, they have substantial potential for climate research, ecological monitoring, urban development, and disaster prevention. Therefore, it is necessary to fully evaluate their performance over the region. In this study, the performance of two ERA5 (ERA5-Land and ERA5-HRES), and three IMERG (IMERG ER, IMERG LR, and IMERG FR) products, as well as their respective predecessors (ERA-Interim and TMPA-3B42) were evaluated comprehensively based on the GSOD ground-based daily observations, from 2001 to 2018 on the Mongolian Plateau. The evaluation was conducted in terms of characterization of precipitation patterns, as well as estimation accuracy and detection ability for daily precipitation. The primary conclusions can be summarized as follows:

1. With respect to precipitation patterns, all seven datasets broadly reproduced seasonal cycles and spatial distributions within their respective spatial coverage. However, only ERA5-HRES, ERA5-Land, IMERG FR, ERA-Interim, and TMPA-3B42 could accurately capture interannual and decadal variability, showing significant correlation coefficients with observed variability at more than 50% of the stations.
2. Regarding estimating and detecting daily precipitation, the new generation of products provided more accurate precipitation information than their predecessors. The overall performance of the evaluated datasets ranked ERA5-Land > ERA5-HRES > ERA-Interim > IMERG FR > IMERG LR > IMERG ER > TMPA-3B42, as shown by their overall $Corr$ (0.55, 0.50, 0.49, 0.41, 0.37, 0.36, and 0.33, respectively) and $ETS$ (0.33, 0.31, 0.28, 0.22, 0.20, 0.20, and 0.18, respectively) scores.
3. The assessed satellite and reanalysis products performed better in the summer ($Corr$ and $ETS$ respectively range 0.60–0.63 and 0.19–0.30) than in the winter ($Corr$ and $ETS$ respectively range 0.42–0.48 and 0.02–0.28), likely due to the solid winter precipitation that is difficult to characterize.
4. All products overestimated weak precipitation ($RB$ ranges 0.21–9.86% at < 2 mm/d) and underestimated high-intensity precipitation ($RB$ ranges − 0.86% to − 0.29% at > 10 mm/d), which led to the better characterization of moderate-intensity precipitation. This suggests that the current ERA5 and IMERG are not the best solution for analysis of extreme events, but have better application potential for hydrological simulation, soil moisture estimation and agricultural management.
5. Topographic complexity showed a negative effect on the detection capability of the satellite IMERG and TMPA products, as evidenced by their significant $ETS$ decreasing trends of − 0.0029 to − 0.0038 versus slope.
6. Affected by different precipitation intensities across the study area, the products generally performed best in agricultural lands and forests along the northern and south-eastern edges ($Corr$ and $ETS$ respectively range 0.34–0.63 and 0.17–0.37), second best in urban areas and grasslands closer to the center ($Corr$ and $ETS$ respectively range 0.30–0.57 and 0.17–0.35), and worst in sparse vegetation and bare areas to the south-west ($Corr$ and $ETS$ respectively range 0.22–0.50 and 0.13–0.30). Due to the topographic effects, IMERG displayed poor detection capabilities in forests (with $ETS$ in the range of 0.17–0.18).

In conclusion, ERA5 generally showed superior performance compared to IMERG over the arid Mongolian Plateau with complex topography. However, each of the ERA5 and IMERG products has its own strengths and limitations. ERA5-HRES could capture long-term precipitation variability, but is limited by the coarse spatial resolution, thus being suitable for long-term large-scale studies. With fine spatial resolution and better characterization of daily precipitation, ERA5-Land can be used for research on glacio-hydrology and topographic precipitation detection, albeit with the overall overestimation. IMERG FR showed deficiencies in representing daily precipitation, but provided more accurate monthly estimates, which make it applicable to hydrological processes studies at seasonal scales. With short release delay and fine resolutions, IMERG ER has great potential for real-time applications such as flood warning, but still needs much improvements in its estimation and detection capability. This evaluation provides a comprehensive understanding of the performance of the newly released ERA5 and IMERG products, which may be a useful reference for research and application data selection.

## Supplementary Information

Supplementary Figures.

## Data Availability

The ground-based observations can be accessed through the National Centers for Environmental Information data website (https://www.ncei.noaa.gov/access/search/data-search/global-summary-of-the-day). The TMPA-3B42 data are available through the Goddard Earth Sciences Data and Information Services Center website (https://disc.gsfc.nasa.gov/). The IMERG products can be found at the Precipitation Measurement Missions (PMM) website (https://gpm.nasa.gov/data/directory). The ERA-Interim data can be acquired from the European Centre for Medium-range Weather Forecasts website (http://apps.ecmwf.int/datasets/). The ERA5 datasets are available on the Climate Data Store (https://cds.climate.copernicus.eu/). The DEM data can be downloaded from the Land Processes Distributed Active Archive Center website (https://lpdaac.usgs.gov/products/astgtmv003/#tools). The land cover data can be acquired from the European Space Agency Climate ChangeInitiative‐Land Cover website (http://maps.elie.ucl.ac.be/CCI/viewer/download.php).
