# Supplementary material for: Evaluation of IMERG and ERA5 precipitation products over the Mongolian Plateau
Source: Sci Rep. 2022 Dec 16;12:21776. doi: 10.1038/s41598-022-26047-8 (PMC9758237; doi:10.1038/s41598-022-26047-8)
Supplement: Supplementary file 1 — Supplementary Figures. [file 41598_2022_26047_MOESM1_ESM.docx]

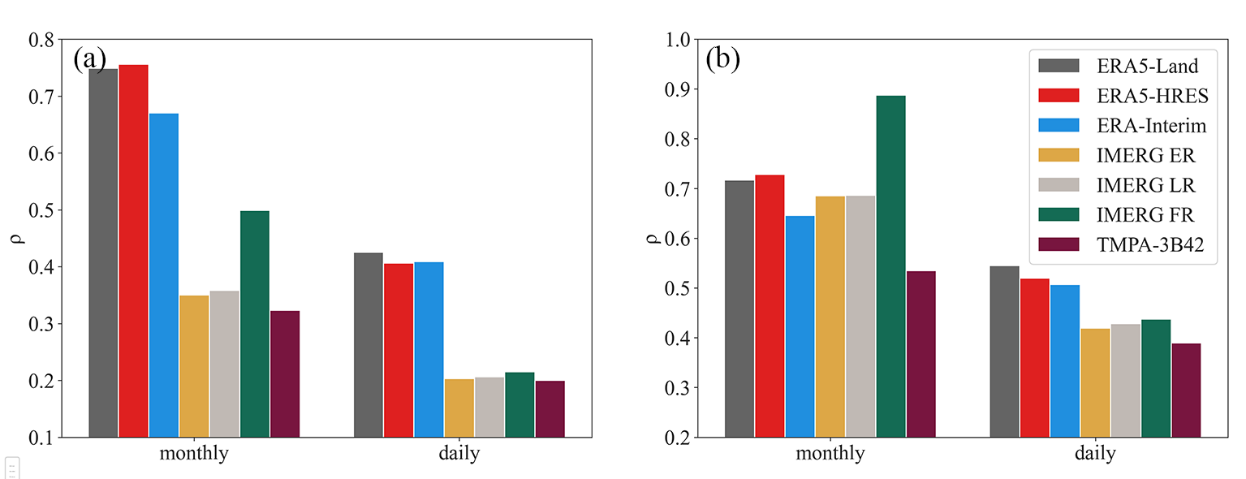
Figure S1. Average spatial spearman correlation coefficients for each product at different temporal scales in (a) winter, and (b) summer.


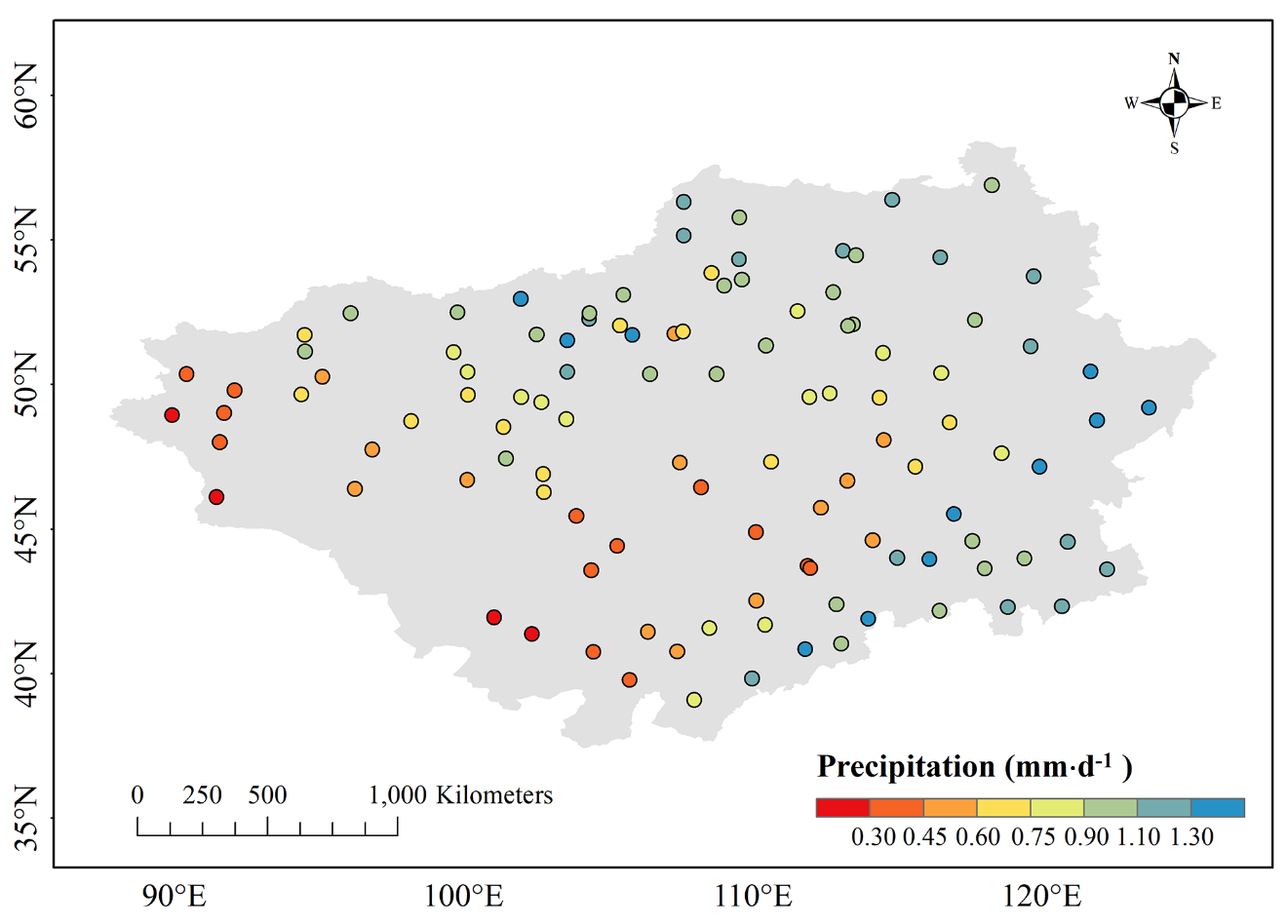
Figure S2. Spatial distribution of multi-year average daily precipitation observed at each station on the Mongolian Plateau from 2001 to 2018. The figure was created using ArcGIS 10.7 (https://www.esri.com/en-us/arcgis/about-arcgis/overview).


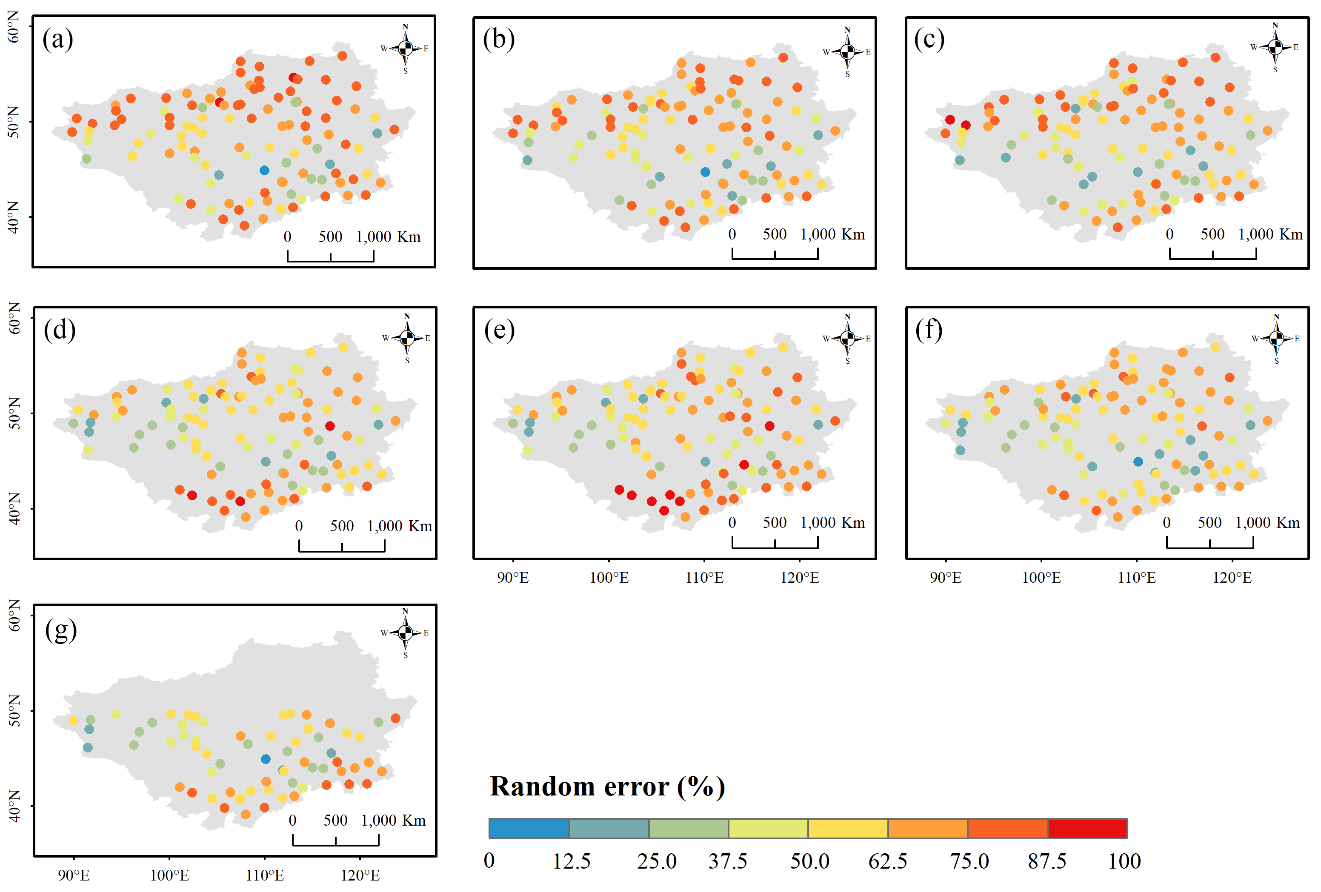
Figure S3. Spatial distribution of random errors for (a) ERA5-Land, (b) ERA5-HRES, (c) ERA-Interim, (d) IMERG ER, (e) IMERG LR, (f) IMERG FR, and (g) TMPA-3B42 from 2001 to 2018. For subplot (g), we only plot the stations within the spatial coverage of TMPA-3B42 (50°N–50°S). The figure was created using ArcGIS 10.7 (https://www.esri.com/en-us/arcgis/about-arcgis/overview).


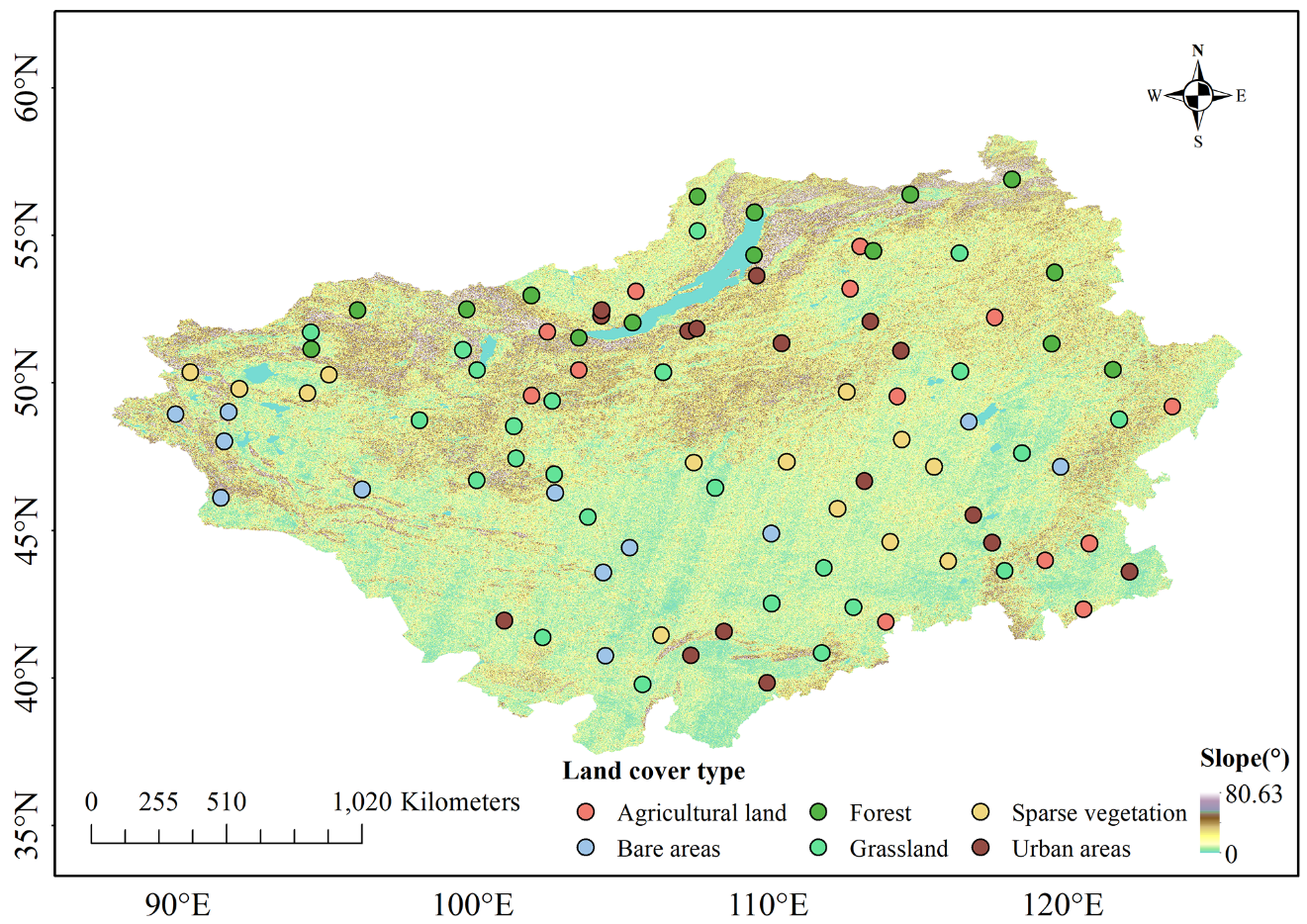
Figure S4. Stations with no change in land cover type mapped on the slope of the study area. The figure was created using ArcGIS 10.7 (https://www.esri.com/en-us/arcgis/about-arcgis/overview).
